# Combined transcriptome and metabolome analysis of *Nerium indicum* L. elaborates the key pathways that are activated in response to witches’ broom disease

**DOI:** 10.1186/s12870-022-03672-z

**Published:** 2022-06-14

**Authors:** Shengjie Wang, Shengkun Wang, Ming Li, Yuhang Su, Zhan Sun, Haibin Ma

**Affiliations:** The Key Laboratory of National Forestry and Grassland Administration for Tropical Forestry Research, Research Institute of Tropical Forestry, Chinese Academy of Forestry, Longdong, Guangzhou, 510520 China

**Keywords:** Plant-pathogen interaction, Defense responses, MAPK-signaling cascade, Linoleic acid, Phenolic acids, Lignans and coumarins, Nerium spp

## Abstract

**Background:**

*Nerium indicum* Mill. is an ornamental plant that is found in parks, riversides, lakesides, and scenic areas in China and other parts of the world. Our recent survey indicated the prevalence of witches’ broom disease (WBD) in Guangdong, China. To find out the possible defense strategies against WBD, we performed a MiSeq based ITS sequencing to identify the possible casual organism, then did a de novo transcriptome sequencing and metabolome profiling in the phloem and stem tip of *N. indicum* plants suffering from WBD compared to healthy ones.

**Results:**

The survey showed that Wengyuen county and Zengcheng district had the highest disease incidence rates. The most prevalent microbial species in the diseased tissues was *Cophinforma mamane*. The transcriptome sequencing resulted in the identification of 191,224 unigenes of which 142,396 could be annotated. There were 19,031 and 13,284 differentially expressed genes (DEGs) between diseased phloem (NOWP) and healthy phloem (NOHP), and diseased stem (NOWS) and healthy stem (NOHS), respectively. The DEGs were enriched in MAPK-signaling (plant), plant-pathogen interaction, plant-hormone signal transduction, phenylpropanoid and flavonoid biosynthesis, linoleic acid and α-linoleic acid metabolism pathways. Particularly, we found that *N. indicum* plants activated the phytohormone signaling, MAPK-signaling cascade, defense related proteins, and the biosynthesis of phenylpropanoids and flavonoids as defense responses to the pathogenic infection. The metabolome profiling identified 586 metabolites of which 386 and 324 metabolites were differentially accumulated in NOHP vs NOWP and NOHS and NOWS, respectively. The differential accumulation of metabolites related to phytohormone signaling, linoleic acid metabolism, phenylpropanoid and flavonoid biosynthesis, nicotinate and nicotinamide metabolism, and citrate cycle was observed, indicating the role of these pathways in defense responses against the pathogenic infection.

**Conclusion:**

Our results showed that Guangdong province has a high incidence of WBD in most of the surveyed areas. *C. mamane* is suspected to be the causing pathogen of WBD in *N. indicum. N. indicum* initiated the MAPK-signaling cascade and phytohormone signaling, leading to the activation of pathogen-associated molecular patterns and hypersensitive response. Furthermore, *N. indicum* accumulated high concentrations of phenolic acids, coumarins and lignans, and flavonoids under WBD. These results provide scientific tools for the formulation of control strategies of WBD in *N. indicum*.

**Supplementary Information:**

The online version contains supplementary material available at 10.1186/s12870-022-03672-z.

## Background

*Nerium indicum* Mill. is a large upright evergreen shrub which belongs to family *Apocynaceae*. It is found all over the world (naturalized in tropical, sub-tropical, and temperate parts) especially in south-west Asia. In particular, it is native to Bangladesh, China, India, Nepal, Myanmar, and Pakistan [1]. In China it is distributed as far as Yunnan and is mostly used as an ornamental plant. The use as an ornamental plant is due to its large gorgeous flowers. This is why it is found in scenic areas, roadsides, riversides, parks, and lakesides. This acclimatization to different growing conditions is also due to its ability to detoxify, absorb, and tolerate pollution such as automobile exhaust [2]. Other than ornamental uses, it is known for the presence of bioactive compounds having medicinal importance [1, 3]. Thus, this species has aesthetic, environmental, and medicinal importance and should be protected from different biotic and abiotic stresses [4].

One of the most devastating disease in *Nerium* species is the witches’ broom disease (WBD) [5]. Typical WBD symptoms include auxiliary bud sprouting (abnormal brush like cluster of dwarfed weak shoots), internode shortening, base thickening, and leaf yellowing. The diseased plants assume stunted growth [5]. Other than *Nerium* spp. this disease has also been reported in a number of plant species such as Mexican/acid lime (*Citrus aurantifolia*) [6, 7], cacao (*Theobroma cacao*), sesame (*Sesamum indicum*) [8], paulownia (*Paulownia tomentosa*) [9], and *Balanites trifloral* [10]. Research on different plant species have shown that the infection causes hypertrophy and hyperplasia in the diseased tissues followed by the loss of apical dominance. Infection leads to the development of abnormal stems called as green broom. In the latter stages, it causes necrosis and death of the diseased tissues [11].

Different plants have been explored to study the resistance mechanism and defense responses against WBD. For example, in cacao, during the development of WBD, Scarpari et al., [12] have reported the changes in the contents of soluble sugars, asparagine and alkaloids, ethylene, and tannins. The authors reported a coordinated biochemical response to the pathogen’s infection in cacao [12]. The transcriptome profiling of the *Paulownia* spp. Suffering from WBD showed the involvement of Ca^2+^ signaling, plant-pathogen interaction pathway, phosphorylation cascade, photosynthesis, and carbohydrate metabolism pathways [13]. In Mexican lime, the whole metabolome profiling during WBD progression resulted in the differential accumulation of 40 different metabolites such as alcohols, organic acids, fatty acids, and amino acids [14]. Another study in Mexican lime indicated the increased levels of catechin and epicatechin together with the higher expression of enzymes related with the phenylpropanoid biosynthesis pathway [15]. The transcriptome sequencing of the same lime species indicated that plants respond to WBD by activating plant-pathogen interaction pathway, along with the changes in cell wall biosynthesis and degradation, regulation of the hormone signaling, and sugars related pathways [6]. Soybean (*Glycine max*) initiates (in response to the infection) an array of defense responses such as activation of plant-pathogen interaction pathway, auxin, cytokinin, ethylene, salicylic acid (SA), Jasmonic acid (JA), and brassinosteroids signaling [16].

Advancements in transcriptome sequencing and metabolite profiling has geared up the discovery of pathways associated with biotic and abiotic stresses in a variety of plant species [17, 18]. These advancements have been successfully utilized in exploring the plant-pathogen interaction during the WBD in various plant species as described in the above paragraph. However, there is no such report on the question that how *N. indicum* plants respond to the WBD. This combined transcriptome and metabolome study will enable researchers to find possible defense strategies of *N. indicum* plants against WBD and formulate corresponding control measures.

## Results

### Disease incidence in Guangdong, China

**Fig. 1:**
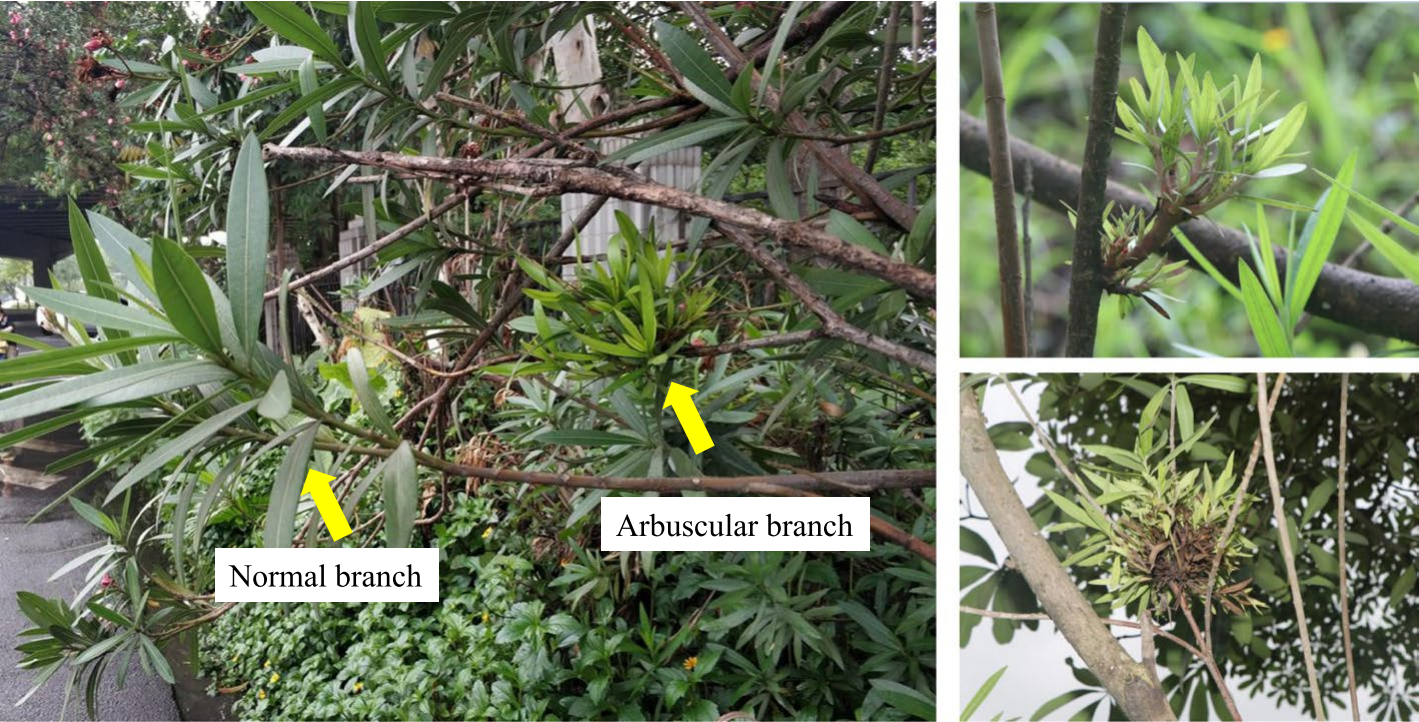
Surveyed N. indicum plants suffering from WBD and showing the characteristic symptoms i.e., normal and arbuscular branches in the larger panel. The smaller panels show arbuscular branches

A survey in Guangdong province, China identified that Shaoguan city (Wengyuen county) had the highest disease incidence rate (80.56%) followed by the Southwest side of Dongguan city (48.08%) (Table 1; Fig. 1). On the other hand, the disease index was the highest in Guangzhou city (Zengcheng district, 71.67%) followed by Shaoguan city (Wengyuen county, 44.33%). Overall, we observed that the disease is more prevalent in rural areas, while the disease incidence was lower in urban highways and park landscapes. It could be noted that there exist correlation between incidence rate and disease index except Zengcheng district and Wengyuen county. This survey report proposes that this disease can impact more than 80% of the plantations in an area. Thus, we further conducted experiments to understand the mechanisms through which *N. indicum* plants respond to WBD.

**Table 1:** Witches’ broom disease incidence in ten different locations of Guangdong province, China

Survey location	Location	Total number of investigated plants	Plants with disease	Incidence rate (%)	Disease index (%)
Dongguan city	In the northeast	635	110	17.32	16.27
	In the southwest	156	75	48.08	41.35
Gaozhou city	Banqiao	290	5	1.72	1.38
Guangzhou city	Yuexiu district	110	0	0.00	0.00
	Fanyu district	126	4	3.17	2.65
	Tianhe district	208	26	12.50	7.37
	Zengcheng district	150	50	33.33	71.67
Huizhou city	Huicheng district	460	0	0.00	0.00
	Huidong county	120	35	29.17	25.00
Jieyang city	Jie dong distric	136	0	0.00	0.00
	Rongcheng district	156	1	0.64	0.01
Meizhou city	Meijiang district	510	0	0.00	0.00
Shenzhen city	Baoan district	89	2	2.25	2.25
	Luohu district	405	0	0.00	0.00
Zhongshan city	Shaxi town	57	5	8.77	8.77
	Tanzhou town	100	0	0.00	0.00
Zhuhai city	Xiangzhou district	767	80	10.43	11.95
	Qiao island	100	0	0.00	0.00
Shaoguan city	Wengyuen county	1260	1015	80.56	44.33

### Community composition and diversity of microorganisms in diseased *N. indicum*

**Fig. 2:**
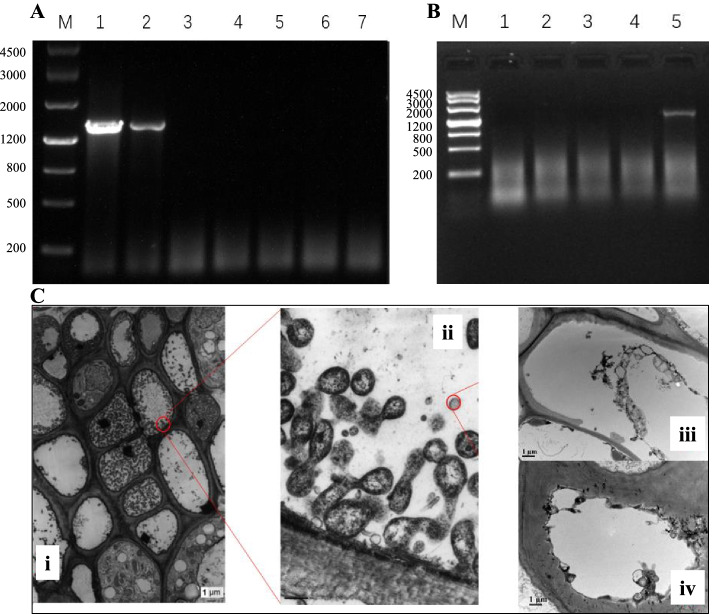
PCR and microscopic analyses of the diseased and healthy *N. indicum* tissues. A) PCR analysis of Jujube (diseased tissues), Paulownia arbuscular diseased tissues, and N. indicum tissues. M: Ladder, 1: Jujube sample, 2: Paulownia arbuscular diseased sample, 3–6: diseased N. indicum, 7: healthy N. indicum. B) Nested PCR detection assay (M: Ladder, 1–4: diseased N. indicum tissues, 5: Paulownia arbuscular diseased tissues). C) Transmission electron microscopy (i, ii: Phytoplasma in the phloem of the deciduous leaf of the Chrysanthemum; iii, iv: phloem of the N. indicum)

First of all, we confirmed if the causal agent of the WB infection in *N. indicum* was phytoplasma or fungi. For this, we tested the presence of phytoplasma because its infection in certain plants such as Jujube, Paulownia, Coconut, and Chrysanthemum have shown symptoms such as yellowing of leaves, clustering and dwarfing of the leaflets, which are sometimes consider similar to those of WBD (Announcement No. 4 of 2013 of the State Forestry Administration). The PCR and nested PCR analyses based on 16S rRNA showed no detection of phytoplasma in the symptomatic *N. indicum* samples (Fig. 2A-B; Supplementary Fig. 1). These PCR assay results were further confirmed by the transmission electron microscopy, where no phytoplasma could be detected (Fig. 2C).

**Fig. 3:**
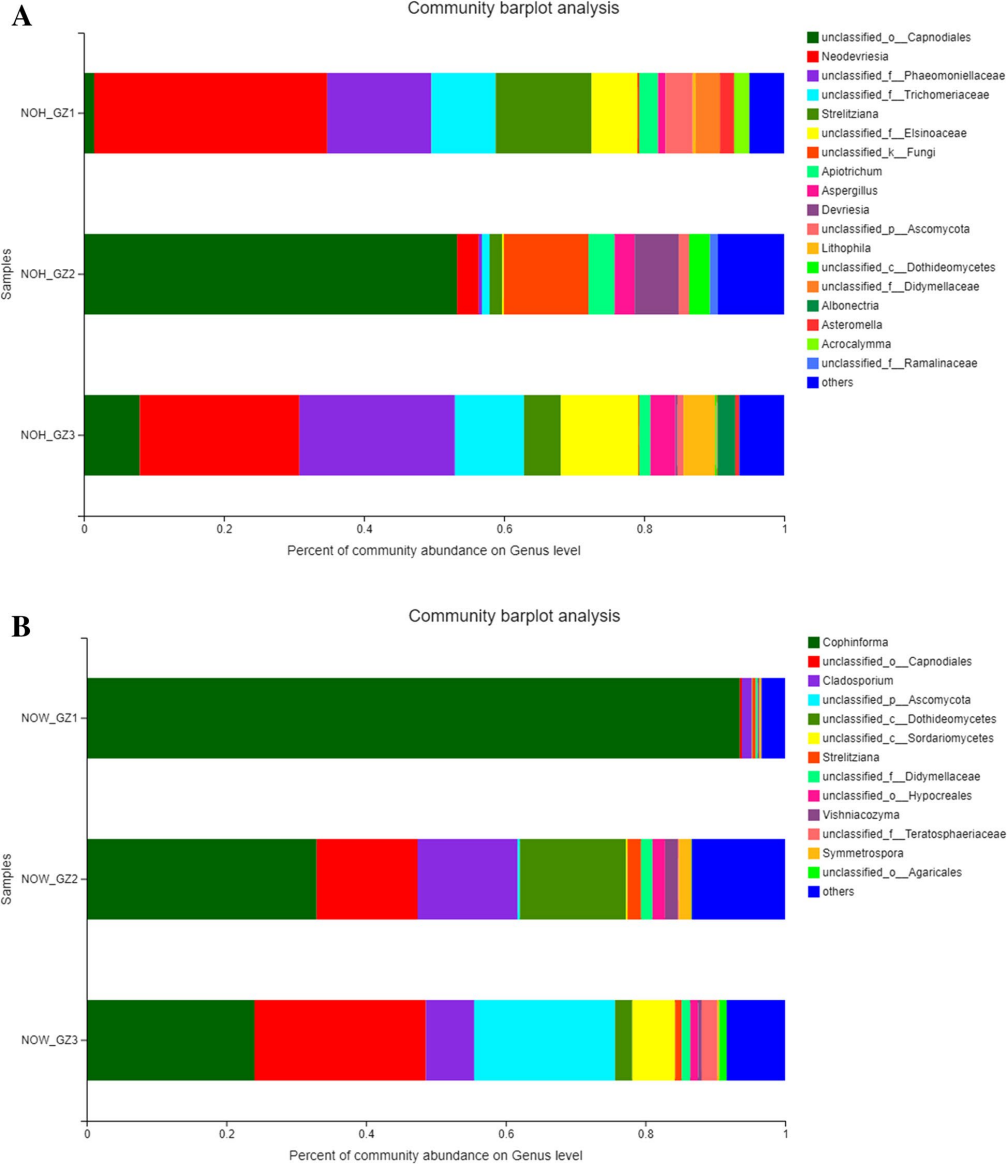
Stacked bar plot showing relative abundances of fungal order in the **A**) healthy and **B**) arbuscular tissues of N. indicum plants. Plots are based on sequencing data for ITS genes. NOH and NOW shows healthy and diseased samples, respectively. The GZ (1–3) with the sample names indicate the replicates

Moving further, we performed MiSeq based 16S rRNA sequencing to identify the casual organism. The sequencing results showed that the average number of bases and sequence length was 17,687,861 bases and 239 bp (min. 153 bp and max. 465 bp), respectively. Alpha diversity analysis showed that the endophytic fungal diversity was higher (Table 2) in the symptomatic stems from Shaoguang, followed by Dongguan, and Guangzhou, which is consistent with the incidence rate. Community composition analysis showed significant differences in endogenous fungal community composition of healthy and diseased *N. indicum* tissues. Among the healthy tissues, the top five fungi with higher abundance were *Capnodiales*, *Neodevriesia*, *Phaeomoniellaceae*, *Trichomeriaceae*, and Strelitziana. Among the *N. indicum* tissues where tufts occurred, the top five fungi with higher abundance were Cophinforma, Capnodiales, Ascomycota, Cladosporium, and Albonectria. The most prevalent microbial species in the healthy samples (NOH) were *Capnodiales neodevriesia* and *Phaeomoniellaceae trichomeriaceae* (Fig. 3A)*.* On the contrary, *Cophinforma mamane* was the most prevalent microbial species in the diseased tissues (Fig. 3B). Interestingly, *C. mamane* was not identified in any healthy tissue, suggesting that this microbial species is the potential pathogen causing WBD in *N. indicum*.

**Table 2:** Diversity index of endophytic fungi

Sample	ace	chao	coverage	shannon	simpson
NOW_DG1	228.92	228.57	1.00	2.74	0.21
NOW_DG2	310.51	308.73	1.00	2.04	0.46
NOW_DG3	247.30	246.44	1.00	2.68	0.22
NOW_GZ1	157.96	156.40	1.00	0.48	0.87
NOW_GZ3	327.81	329.00	1.00	2.89	0.12
NOW_GZ2	275.52	277.00	1.00	2.86	0.16
NOW_SG1	331.26	328.38	1.00	2.58	0.22
NOW_SG2	295.41	294.70	1.00	3.26	0.07
NOW_SG3	263.92	267.18	1.00	2.46	0.21
NOH_GZ1	85.00	85.00	1.00	2.69	0.15
NOH_GZ2	111.03	110.33	1.00	3.51	0.05
NOH_GZ3	110.52	111.00	1.00	2.85	0.12

NOH and NOW shows healthy and diseased samples, respectively. The GZ (1–3) with the sample names indicate the replicates

### Transcriptome sequencing of N. indicum

The sequencing of 12 libraries of *N. indicum* resulted in 42.93 to 51.21 million clean reads (average clean reads per library were 47.07 million reads). Overall, the transcriptome sequencing of *N. indicum* resulted in 86.7 Gb clean data; the average clean data of each sample reached 6 Gb. The error rate was ≤ 0.03%. The Q20%, Q30%, and GC% were ≤ 97.94%, ≤ 93.89%, and 43.38%, respectively (Supplementary Table 1). The transcriptome sequencing resulted in the identification of 191,224 unigenes. Of these, 142,396 unigenes could be annotated in different databases i.e., KEGG (111,585), NR (141,221), SwissProt (102,226), Trembl (139,139), KOG (89,276), GO (119,702), Pfam (107,887) (Supplementary Fig. 2).

**Fig. 4:**
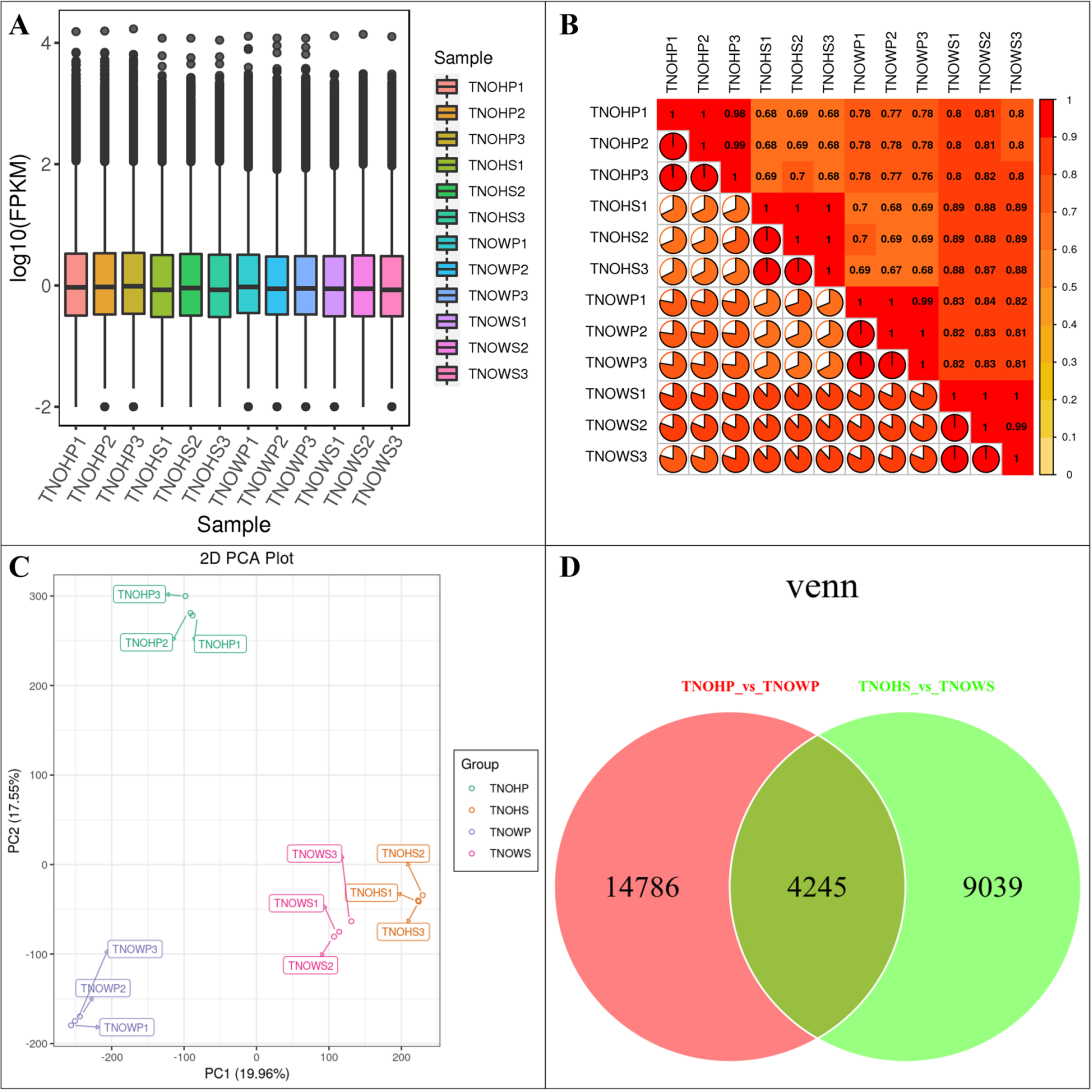
Summary of gene expression in diseased Nerium indicum L. stem tip and phloem and respective controls. **A** Overall distribution of gene expression, **B** Pearson Correlation Coefficient between different treatments and their replicates, **C** Principal component analysis, and **D** Venn diagram of the differentially expressed genes. Where TNOWS, TNOHS, TNOWP, and TNOHP represent diseased stem tip, healthy stem tip, diseased phloem, and healthy phloem of N. indicum. 1, 2, and 3 with the tissue names represent replicates

The overall gene expression distribution i.e., Fragments Per Kilobase of Transcript per Million fragments mapped (FPKM) was lower in healthy stem (NOHS) as compared to the diseased stem (NOWS), diseased phloem (NOWP), and healthy phloem (NOHP) (Fig. 4A). The Pearson Correlation Coefficient (PCC) for the biological and technical replicates was 0.68 to 0.99 (average PCC = 0.8) (Fig. 4B) indicating the reproducibility of the experiment and reliability of the expression data. The 1^st^ and 2^nd^ principal components (PC1 and PC2) explained 19.96% and 17.555% (Fig. 4C). We found 13,284 and 19,031 DEGs in NOHP vs NOWP and NOHS and NOWS, respectively (Fig. 4D; Supplementary Tables 2–3). The top-10 KEGG pathways in which DEGs were significantly enriched (in NOHP vs NOWP) were plant-pathogen interaction, stilbenoid, diarylheptanoid and gingerol biosynthesis, plant hormone signal transduction, phenylpropanoid biosynthesis, linoleic acid metabolism, flavonoid biosynthesis, metabolic pathways, biosynthesis of secondary metabolites, MAPK signaling pathway-plant, and steroid biosynthesis (Supplementary Fig. 3). Whereas in NOHS vs NOWS, the DEGs were significantly enriched in MAPK signaling pathway, plant hormone signal transduction, flavonoid biosynthesis, biosynthesis of secondary metabolites, circadian rhythm, ABC transporters, zeatin biosynthesis, sesquiterpenoid and triterpenoid biosynthesis, plant-pathogen interaction, and alpha-linoleic acid metabolism (Supplementary Fig. 3). From these observations, it could be concluded that the common pathways could be highly relevant to the *N. indicum* responses to the WBD.

Of the 19,031 DEGs between NOHP and NOWP, 562 and 2,330 genes were exclusively expressed in NOHP and NOWP, respectively. The 276 genes that were exclusively expressed in NOHP were enriched in metabolic pathways, starch and sucrose metabolism, biosynthesis of secondary metabolites, and carbon metabolism (Supplementary Fig. 4A). While the 915 genes specific to NOWP were enriched in lysine biosynthesis, citrate cycle, linoleic acid metabolism, biosynthesis of amino acids, plant-pathogen interaction and biosynthesis of secondary metabolites pathways (Supplementary Fig. 4B). The regulation of these pathways in NOWP indicates that *N. indicum* phloem have exclusively regulated these pathways.

Of the 13,284 DEGs between NOHS and NOWS, 215 and 976 genes were specific to NOHS and NOWS, respectively. The genes that were exclusively expressed in the NOHS were enriched in biosynthesis of secondary metabolites and metabolic pathways (Supplementary Fig. 4C). Whereas, the NOWS specific genes were enriched in glycolysis/gluconeogenesis, metabolic pathways, fatty acid degradation, biosynthesis secondary metabolites, amino sugar and nucleotide sugar metabolism, and plant-pathogen interaction pathways (Supplementary Fig. 4D).

### Differential regulation of signaling related pathways

Two major pathways related to signaling i.e., MAPK signaling-plant pathway and plant-hormone signal transduction pathway, were differentially regulated between the diseased and healthy *N. indicum* studied tissues. There were 434 and 545 DEGs enriched in MAPK signaling-plant and plant hormone signal transduction pathways, respectively.

**Fig. 5:**
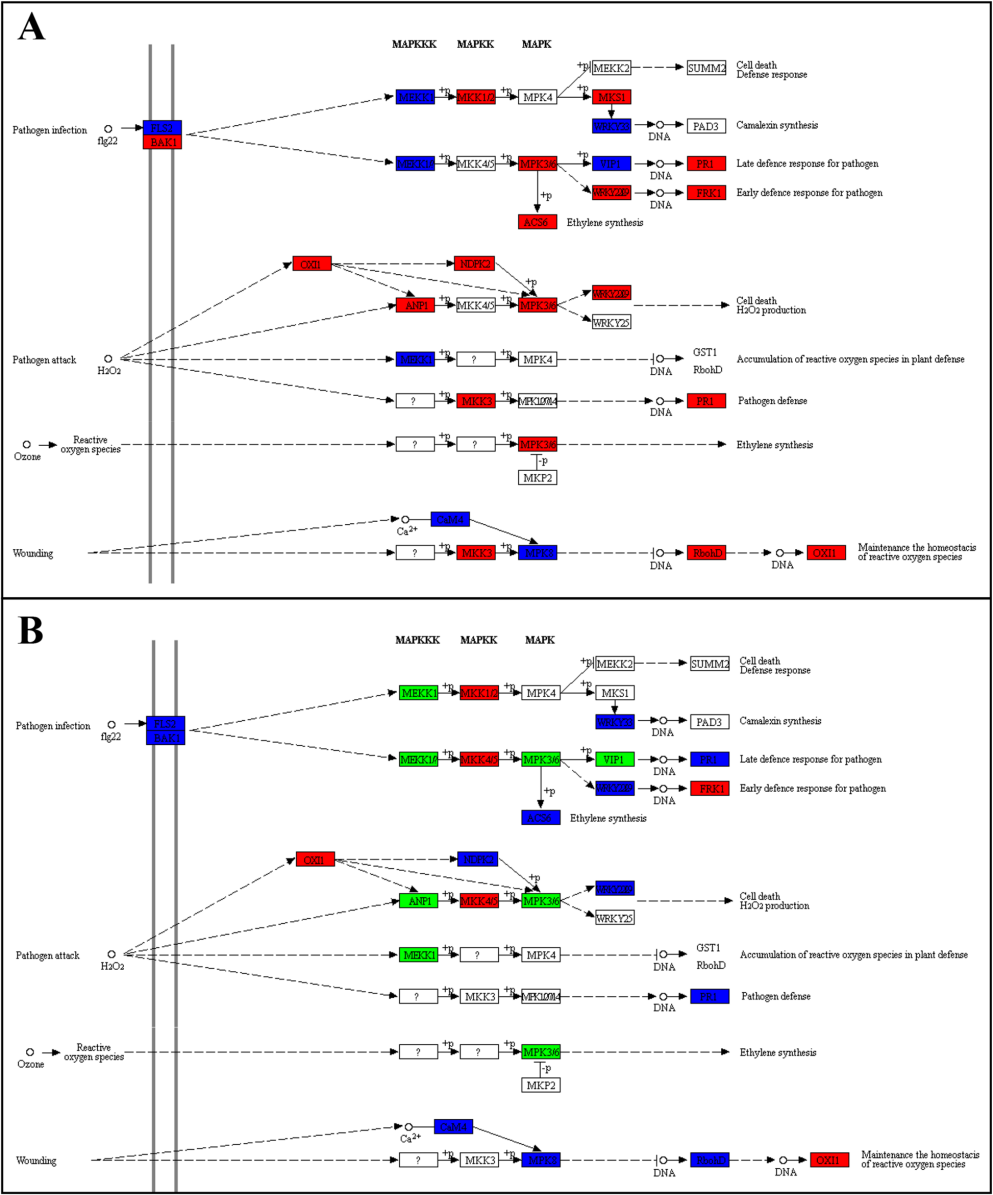
Differential regulation of MAPK signaling-plant pathway in A) NOHP vs NOWP and B) NOHS vs NOWS). The red, green, and blue color indicates increased, decreased, and increased/decreased expression of the respective genes in the given pathways. Where NOWS, NOHS, NOWP, and NOHP represent diseased stem tip, healthy stem tip, diseased phloem, and healthy phloem of N. indicum. Permissions to use the KEGG pathway map was taken from the Kanehisa Laboratories (https://www.kanehisa.jp/)

- MAPK Signaling-plant pathway
  Specifically, in the MAPK signaling-plant pathway, we observed that signaling pathway related to pathogen infection was activated. The key genes i.e., botrytis-induced kinase 1 (BAK1), mitogen-activated protein kinase kinase 1/2 (MKK1/2), MAP-kinase substrate 1 (MKS1), MAP-kinase (MPK), MPK3/6, ACS6, WRKY TF, pathogenesis-related protein 1 (PR1), and FRK1 were upregulated in NOWP as compared to NOHP (Fig. 5A). Whereas in MKK1/2, MKK4/5, and FRK1 were upregulated, while, MEKK1, MPK3/6, and VIP1 were downregulated in NOWS as compared to NOHS (Fig. 5B; Supplementary Table 4). These observations indicate that MKKs, FRK1, and PR1 play important roles in early and late responses to WBD. Furthermore, we found that genes associated with pathogen attack (and H_2_O_2_) were upregulated in WBD. Importantly, we noted the increased expression of OXI1, ANP1, NDPK2, MPK3/6, and WRKY TFs related with cell death and H_2_O_2_ production in NOWP as compared to NOHP. Similarly, the OXI1, MPK3/6, WRKY TF, and NDPK2 showed increased expressions in NOWS as compared to NOHS. Additionally, RobhD was also upregulated in the diseased tissues; RobhD together with MKK3, MPK8, and CaM4 were involved in the maintenance of the homeostasis of reactive oxygen species (ROS) [19, 20] (Fig. 5).
- Plant-hormone signal transduction pathway

**Fig. 6:**
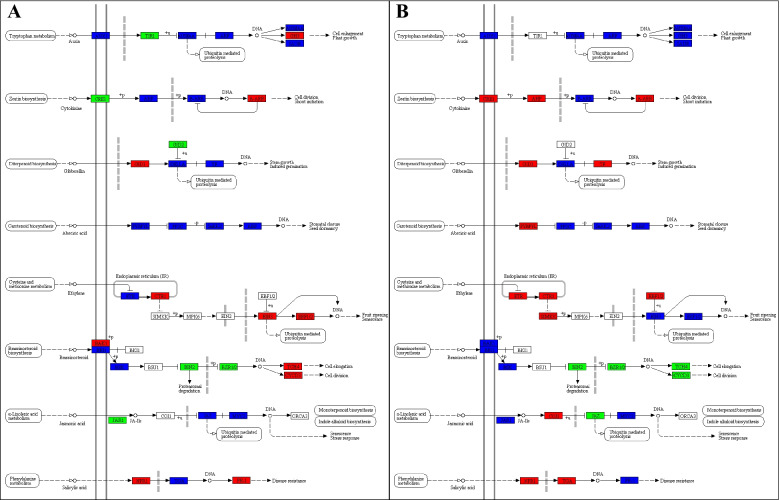
Differential regulation of plant-hormone signal transduction pathway in **A** NOHP vs NOWP and **B** NOHS vs NOWS. The red, green, and blue color indicates increased, decreased, and increased/decreased expression of the highlighted genes. Where NOWS, NOHS, NOWP, and NOHP represent diseased stem tip, healthy stem tip, diseased phloem, and healthy phloem of N. indicum. Permissions to use the KEGG pathway map was taken from the Kanehisa Laboratories (https://www.kanehisa.jp/)

Regarding plant-hormone signal transduction pathway, we noted that almost all hormone signaling related pathways were differentially regulated. For auxin signaling, TRANSPORT INHIBITOR RESPONSE1 (TIR1) and Glycoside hydrolase 3 (GH3) showed decreased and increased expression in NOWP as compared to NOHP, respectively. While the transcripts related to other genes in this pathway showed mixed regulation (some transcripts showed increased expression while other showed decreased expression). In stem, all the genes showed mixed expression. These observations indicate that auxin signaling has minor role in defense against WBD in *N. indicum.* As far as cytokinin signaling is concerned, we noted that CYTOKININ RESPONSE 1 (CRE1) and type-A Arabidopsis response regulator (A-AAR) showed decreased and increased expression, respectively in NOWP as compared to NOHP. Whereas, transcripts related to CRE1, Arabidopsis histidine phosphotransfer proteins (AHP), and A-AAR genes showed higher FPKM values in NOWS as compared to NOHS. This suggests important role of cytokinin in defense against *N. indicum.* For gibberellin signaling, a gene gibberellin insensitive dwarf 1 (GID1) was upregulated in both diseased tissues as compared to their respective control. Whereas GID2 was downregulated in NOWP as compared to NOHP, while TF showed increased expression in NOWS as compared to NOHS. A PYR/PYL gene was upregulated in NOWS as compared to NOHS, while all other genes showed mixed expression in diseased and healthy *N. indicum* phloem and stem (Supplementary Table 4; Fig. 6).

Ethylene signaling was differentially regulated in both tissue types. However, different set of genes were regulated in both tissues e.g., CONSTITUTIVE TRIPLE RESPONSE (CTR1), ETHYLENE INSENSITIVE 3 (EIN3), and ethylene responsive factor 1/2 (ERF1/2) were upregulated in NOWP as compared to NOHP, whereas ethylene receptor (ETR), CTR1, stress induced MAPK kinase kinase (SIMKK), ethylene binding factor 1/2 (EBF1/2) were upregulated in NOWS as compared to NOHS. Transcripts related to other genes in the ethylene signaling were both up/downregulated. The regulation of a large number of ethylene signaling related genes indicates an important role of ethylene signaling defense against WBD in *N. indicum* plants. Three genes i.e., BAK1, touch 4 (TCH4), and cyclin-D3 (CYCD3), were upregulated while two genes i.e., BR Insensitive 2 (BIN2) and BRASSINAZOLE-RESISTANT 1 (BZR1/2) were downregulated NOWP as compared to NOHP. On the contrary, all these genes (except BAK1) were downregulated in NOWS as compared to NOHS. These expression changes suggest that the brassinosteroid signaling is differently regulating the witches’ broom infection/defense in *N. indicum* phloem and stem. Jasmonic acid (JA) signaling related gene JAR1 was downregulated in diseased phloem while CORONATINE INSENSITIVE 1 (COI1) was upregulated in diseased stem as compared to their respective controls. It is known that salicylic acid (SA) plays important roles in plant defense against pathogens by participating in inducible defense mechanism and system acquired resistance (SAR) [21]. In accordance to this, we also observed the differential regulation of the genes involved in SA signaling. Two genes i.e., NONEXPRESSOR OF PATHOGENESIS-RELATED GENES 1 (NPR1) and pathogenesis related protein 1 (PR-1) were upregulated in NOWP as compared to NOHP. Whereas, NPR1 and TF TGA (TGA) showed increased expression in *N. indicum* stem with WBD (Supplementary Table 4; Fig. 6).

These observations suggest that *N. indicum* plants activate a network of signaling mechanisms associated with phytohormones, ROS, and disease resistance against diseased plants with symptoms of witches’ broom in stem and phloem.

### Differential regulation of defense-related pathways

**Fig. 7:**
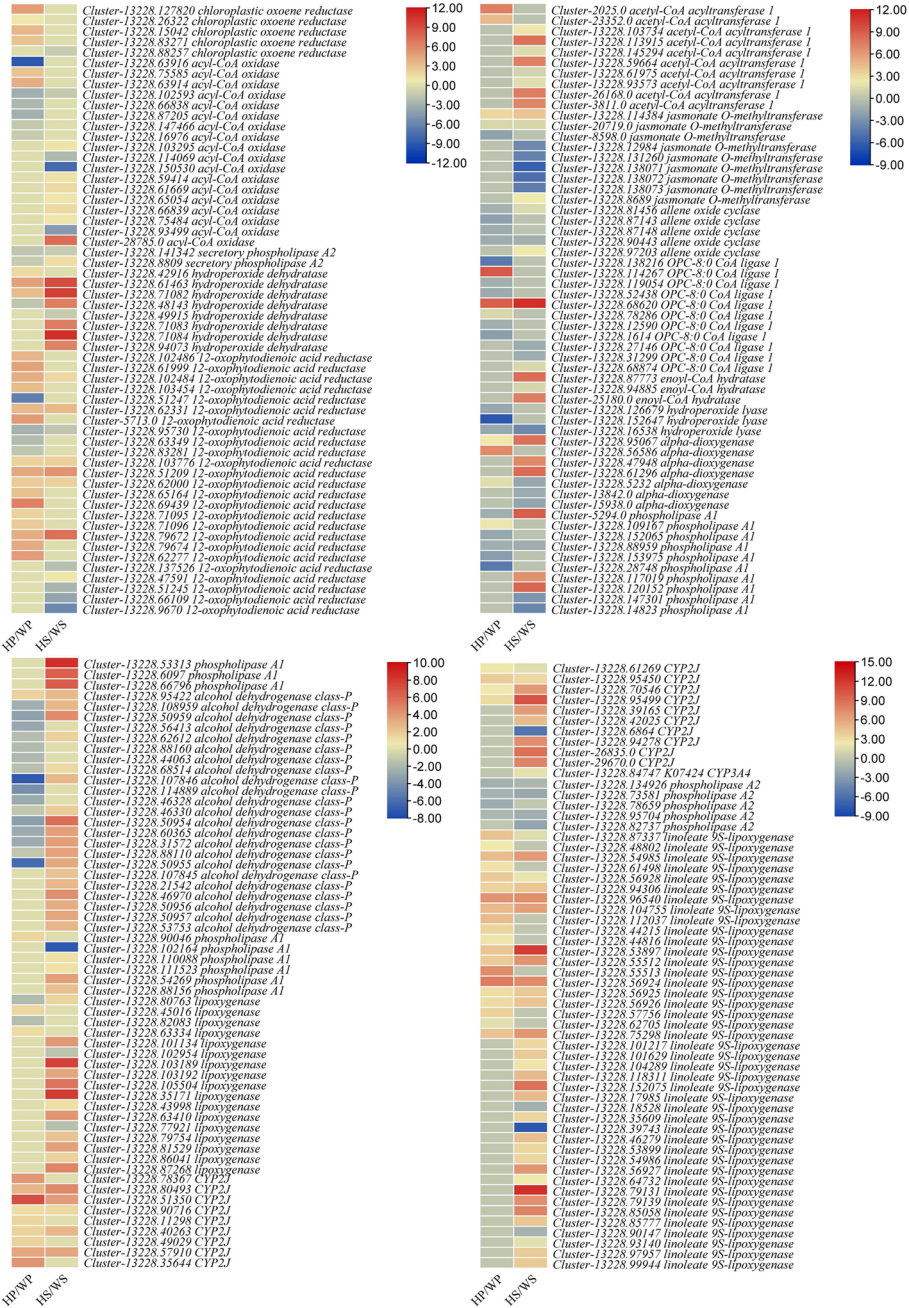
Heatmaps representing the log2 foldchange values of differentially. The gene names are followed by annotation as per KEGG database. Where WS, HS, WP, and HP represent diseased stem tip, healthy stem tip, diseased phloem, and healthy phloem of N. indicum

- Plant-pathogen interaction pathway
  Calcium signaling plays an important role as a universal second messenger in plants’ responses to biotic and abiotic stresses and is a major part of plant-pathogen interaction pathway [22]. In the studied tissue comparisons, we observed the changes in the expression of multiple genes which are associated with plants’ responses (based on Ca^2+^ signaling) against invading pathogens. In NOWP, respiratory burst oxidase (Rboh) was upregulated while nitric-oxide synthase (NOS) was downregulated as compared to NOHP. Whereas, cyclic nucleotide gated channel (CNGCs) were downregulated in NOWS as compared to NOHS. Transcripts related to other genes in the Ca^2+^ signaling were variedly regulated. Other genes that end up in activation of the defense related genes i.e., WRKY TF (especially WRKY29), FLG22-induced receptor-like kinase 1 (FRK1), glycerol kinase (NHO1), and PR-1 showed increased expression in witches’ broom diseased tissues. For example, we observed BAK1/BKK1, pto-interacting protein 5 (PTI5), PTI6, MAPK-kinase kinase 1/2 (MKK1/2), and WRKY TFs were upregulated in NOWP, whereas, EF-TU receptor (EFR1) and MKK4/5 were upregulated in NOWS. All of the four defense related genes i.e., WRKY, FRK1, NHO1, and PR-1 were upregulated in NOWP, whereas only WRKY and FRK1 were upregulated in NOWS in comparison to their respective controls. Transcripts related to cysteine and histidine-rich domain-containing protein RAR1 (RAR1) and enhanced disease susceptibility 1 protein (EDS1) showed increased expression in NOWP as compared to NOHS. The EDS1 was also upregulated in NOWS as compared to NOHS. The diseased stem also showed increased expression disease-resistance protein 2 (RPS2). These expression changes suggest that *N. indicum* plants defend themselves from the witches’ broom infection by activating RAR1 and EDS1 driven responses (Supplementary Table 4). Overall, these observations indicate that *N. indicum* plants adapt multiple level defense strategy to resist the Withes’ broom infection.
- Linoleic acid and α-linoleic acid metabolism pathways
  Eighty-six DEGs were enriched in linoleic acid metabolism pathway; 43 and 70 genes in this pathway were differentially expressed in NOHP vs NOWP and NOHS vs NOWS, respectively. All the transcripts annotated as linoleic acid metabolism related genes were variedly expressed between NOHP and NOWP; secretory phospholipases A2, linoleate 9S-lipoxygenases, cytochrome P450 family 2 subfamily J (CYP2J), and lipoxygenases. However, the transcripts annotated as cytochrome P450 family 3 subfamily A4 (CYP3A4, *Cluster-13228.84747*) showed increased expression in the diseased phloem as compared to control. In addition to CYP2J, we also observed increased expression of linoleate 9S-lipoxygenases in NOWS as compared to NOHS. Whereas, secretory phospholipases 2 were downregulated in diseased stem as compared to control. These observations suggest that the biosynthesis of linoleic acid is not stable, whereas its degradation/breakdown increases in NOWP as compared to NOHP. On the contrary, the biosynthesis of linoleic acid decreased whereas its breakdown increased in NOWS as compared to NOHS (Supplementary Table 4; Fig. 7). We also observed the increased expression of chloroplastic oxoene reductase (COR), acetyl-CoA acyltransferase 1, and phospholipase A1 in NOWP as compared to NOHP. Whereas, in NOWS, only acetyl-CoA acyltransferase 1 related transcripts showed increased expression (Supplementary Table 4; Fig. 7). These changes propose that *N. indicum* plants may adapt JA induced defense system by changing linoleic acid levels.
- Phenylpropanoid and flavonoid biosynthesis pathways
  Since DEGs were significantly enriched in phenylpropanoid biosynthesis pathway and flavonoid biosynthesis pathway, therefore, we explored the differential regulation of these pathways in witches’ broom diseased *N. indicum* plants as compared to healthy controls. There were 355 and 115 DEGs that were enriched in phenylpropanoid biosynthesis and flavonoid biosynthesis pathways, respectively (Supplementary Table 4). Phenylpropanoid biosynthesis was largely affected. Most prominently, we noticed that the expression of trans-cinnamate 4-monooxygenase, caffeoyl-CoA O-methyltransferase (CCOAOMT1), 4-coumarate-CoA ligase (4CL), catalase-peroxidase (Kat), cinnamoyl-CoA reductase (CCR), ferulate-5-hydroxylase (F5H), phenylalanine ammonia-lyase (PAL), caffeoylshikimate esterase (CSE), and cinnamyl-alcohol dehydrogenase (CAD) increased in NOWP as compared to NOHP. Whereas, other genes showed varied expression pattern between NOWP and NOHP. While, coniferyl-alcohol glucosyltransferase was downregulated in NOWP as compared to NOHP. Whereas in NOWS, we observed the increased expression of trans-cinnamate 4-monooxygenase, CCOAOMT1, flavin prenyltransferase, Kat, coumaroylquinate(coumaroylshikimate) 3'-monooxygenase, F5H, PAL, CAD, CSE, and scopoletin glucosyltransferase (SGtf) (Supplementary Table 4).
  From these observations it could be noted that possibly the biosynthesis of caffeoyl-CoA and coniferyl aldehyde increased in NOWP and NOWS which might lead the increased biosynthesis of lignin increased as compared to NOHP and NOHS. Also, it could be suggested that *N. indicum* stem biosynthesize lignin, syringin, coniferin, and 4-hydroxycinnamyl-alcohol-4-D-glucoside as a response against witches’ broom infection. While, phloem of diseased plants only biosynthesizes lignin as compared to the healthy phloem (Supplementary Table 4).
  Most interesting finding was that all the transcripts related to genes associated with flavonoid biosynthesis pathway showed increased expression in NOWP and NOWS as compared to their respective controls. Only one gene (leucoanthocyanidin reductase, *Cluster-13228.75100*) showed reduced expression in NOWS as compared to NOHS (Supplementary Table 4). These expression changes suggest that large scale flavonoid biosynthesis is either initiated or ongoing as a defense response when *N. indicum* plants are presenting WBD symptoms.
- Top DEGs with increased/decreased expression in diseased *N. indicum* phloem and stem
  The top-10 genes with highest log2 fold change values in the diseased phloem and stem as compared to their respective controls are shown in Tables 2–3. The highest log2 foldchange value (13.97) was observed in NOWP for *Cluster-13228.51868* (transcriptional activator of proteases prtt). This exclusive expression in NOWP as compared to NOHP suggests that proteolysis of proteins is highly increased in NOWP and a supply of amino acids is increased. This is consistent with the KEGG pathway enrichment results that DEGs were also enriched amino acid biosynthesis pathway. Since Regulatory Particle Non-ATPase 4 (RPN4) is required for proteolysis, the exclusive expression of transcriptional regulator RPN4 proposes (*Cluster-13228.57048*) that in NOWP, there is proteolysis. Thus, it is possible that the Withes’ broom diseased *N. indicum* plants opt for proteolysis for higher amino acid supplies. This is consistent with the known fact that proteases act as hubs in plant immunity and the function of proteases and related genes [23–25]. Other prominent genes that were exclusively expressed in NOWP included ubiquitin carboxyl-terminal hydrolase, β-amyrin 28-monooxygenase, viridiflorene synthase, abscisic-aldehyde oxidase, cytokinin dehydrogenase, cathepsin A, arrestin-related trafficking adapter, and expansin. The carboxyl-terminal hydrolase (*Cluster-13228.83464*) is a part of circadian clock [26], whereas β-amyrin 28-monooxygenase (*Cluster-13228.51250*) and viridiflorene synthase (*Cluster-13228.74406*) are involved in triterpene and sesquiterpene biosynthesis [27]. The expression of abscisic-aldehyde oxidase (*Cluster-13228.74406*), cytokinin dehydrogenase (*Cluster-13228.56658*), and cathepsin A (*Cluster-13228.110860*) indicate the possible role of ABA biosynthesis [28], zeatin biosynthesis [29], and Jasmonic acid induced defense response [30] in diseased phloem, respectively. This observation is consistent with the KEGG pathways annotation results where we observed the differential regulation of both plant-hormone signal transduction pathway and zeatin biosynthesis pathway (Supplementary Fig. 4). Finally, the exclusive expression of expansin (*Cluster-13228.65339*) (Table 2).
  The highest downregulation of genes in NOWP as compared to NOHP indicates that the processes such as ribosome functioning (*Cluster-13228.67277*), disturbance in photosynthesis possibly due to disturbances in linear electron flow by PROTON GRADIENT REGULATION 5 (*Cluster-13228.107206*) [31], maintenance of genome stability by protein downstream neighbor of Son (Cluster-13228.90657) [32], degradation of arginine to urea by arginase (*Cluster-13228.124673*) [33], disturbance in auxin redistribution in response to gravity by LAZY (*Cluster-13228.124659*) [34], and thiamine metabolism (*Cluster-13228.58619*) [35] were significantly affected in the diseased phloem (Table 3).
  The genes that were highly expressed in NOWS as compared to NOHS were related to transition from vegetative to reproductive phase (lysine-specific histone demethylase 1A, *Cluster-13228.98726*) [36], MAPK-signaling (MAPKKK13, *Cluster-13228.81744*) [37], sucrose related pathways (fructose-bisphosphate aldolase, *Cluster-13228.71255 *[38] and GDP mannose 4,6-dehydratase, *Cluster-13228.55109*) [39], programmed cell death (KDEL-tailed cysteine endopeptidase, *Cluster-13228.81725*), secondary metabolite biosynthesis (tyrosine aminotransferase, *Cluster-13228.107437*), and disease resistance (WRKY19, *Cluster-13228.71611*). These genes were enriched in plant-pathogen interaction, sucrose metabolism related pathways, MAPK-signaling plant, and secondary metabolite biosynthesis, which is consistent with the KEGG pathway enrichments results. Indicating and confirming the involvement of these pathways in resistance against WBD in *N. indicum* stem (Table 4).
  The highly downregulated genes included heat shock protein, peroxin-5, LIFEGUARD4, ribulose-phosphate 3-epimerase, reversibly glycosylated polypeptide / UDP-arabinopyranose mutase, tetraspanin-4, and some other genes. These genes are associated with plant-pathogen interactions e.g., knockdown of LIFEGUARD4 supports delayed fungal development in Arabidopsis [40], pentose phosphate pathway or xylose metabolism [41], and interaction with pathogens [42] (Table 4).
- qRT-PCR analysis of selected genes

**Table 3:** List of top-10 genes that were up/downregulated in diseased *N. indicum* phloem as compared to healthy phloem

Gene ID	FPKM Values		Log2 foldchange	Annotation
	NOHP	NOWP	NOHP/NOWP	
*Cluster-13228.51868*	0.00	47.41	13.97	transcriptional activator of proteases prtt
*Cluster-13228.57048*	0.00	32.70	13.66	transcriptional regulator rpn4
*Cluster-13228.83464*	0.00	17.89	13.62	ubiquitin carboxyl-terminal hydrolase 36/42
*Cluster-13228.51250*	0.00	80.97	13.46	beta-amyrin 28-monooxygenase
*Cluster-13228.74406*	0.00	19.59	13.31	abscisic-aldehyde oxidase
*Cluster-13228.41301*	0.00	51.54	13.27	viridiflorene synthase
*Cluster-13228.56658*	0.00	40.72	13.26	cytokinin dehydrogenase
*Cluster-13228.110860*	0.00	18.56	13.07	cathepsin A (carboxypeptidase C)
*Cluster-13228.119834*	0.00	29.84	13.02	arrestin-related trafficking adapter 4/5/7
*Cluster-13228.65339*	0	594.00	12.91	expansin
*Cluster-13228.124673*	2.37	0.00	-8.16	arginase
*Cluster-13228.124659*	64.11	0.19	-8.35	AtLAZY1
*Cluster-13228.58619*	361.92	0.97	-8.46	thiamine thiazole synthase
*Cluster-13239.0*	4.87	0.01	-8.49	4-hydroxyphenylpyruvate dioxygenase
*Cluster-13228.12868*	74.88	0.19	-8.52	steroid 17alpha-monooxygenase / 17alpha-hydroxyprogesterone deacetylase
*Cluster-13228.104385*	1.09	0.00	-8.57	E3 ubiquitin-protein ligase MGRN1
*Cluster-13228.90657*	1.50	0.00	-8.66	protein downstream neighbor of Son
*Cluster-13228.107206*	39.72	0.11	-8.68	PROTON GRADIENT REGULATION 5
*Cluster-13228.125163*	33.23	0.07	-8.72	charged multivesicular body protein 5
*Cluster-13228.67277*	3.62	0.00	-9.61	ATP-dependent RNA/DNA helicase IGHMBP2

NOWS, NOHS, NOWP, and NOHP represent diseased stem tip, healthy stem tip, diseased phloem, and healthy phloem of *N. indicum*

**Table 4:** List of genes top-10 genes that were up/downregulated in diseased *N. indicum* stem as compared to healthy stem

Gene ID	FPKM values		Log2 foldchange	Annotation
	NOHS	NOWS	NOHS/NOWS	
*Cluster-13228.98726*	0.00	4.43	12.38	lysine-specific histone demethylase 1A
*Cluster-13228.61840*	0.00	55.64	12.24	Not-annotated in any database
*Cluster-13228.83721*	0.00	37.82	11.92	Not-annotated in any database
*Cluster-13228.81744*	0.00	4.97	11.79	mitogen-activated protein kinase kinase kinase 13
*Cluster-13228.71255*	0.00	4.06	11.68	fructose-bisphosphate aldolase
*Cluster-13228.55109*	0.00	9.09	11.38	GDPmannose 4,6-dehydratase
*Cluster-13228.81725*	0.00	21.30	11.20	KDEL-tailed cysteine endopeptidase
*Cluster-13228.107437*	0.00	10.22	11.05	tyrosine aminotransferase
*Cluster-13228.71611*	0.00	7.23	10.86	WRKY transcription factor 19
*Cluster-13228.48102*	0.00	2.87	10.77	Pancreatic ribonuclease
*Cluster-13228.155066*	1.03	0.00	-7.83	tryptophan synthase beta chain
*Cluster-13228.107425*	0.79	0.00	-7.87	trichothecene 3-O-acetyltransferase
*Cluster-13228.63605*	1.30	0.01	-8.02	cyclin-dependent kinase 12/13
*Cluster-13228.59030*	8.04	0.03	-8.03	tetraspanin-4
*Cluster-13228.133143*	1.51	0.00	-8.43	reversibly glycosylated polypeptide / UDP-arabinopyranose mutase
*Cluster-13228.101103*	5.01	0.01	-8.70	ribulose-phosphate 3-epimerase
*Cluster-13228.76336*	1.81	0.00	-8.95	peroxin-5
*Cluster-13228.71456*	13.23	0.02	-9.18	LIFEGUARD 4
*Cluster-13228.97191*	2.17	0.00	-9.23	peroxin-5
*Cluster-13228.76679*	2.65	0.00	-9.31	DnaJ homolog subfamily B member 5

NOWS, NOHS, NOWP, and NOHP represent diseased stem tip, healthy stem tip, diseased phloem, and healthy phloem of *N. indicum*

**Fig. 8:**
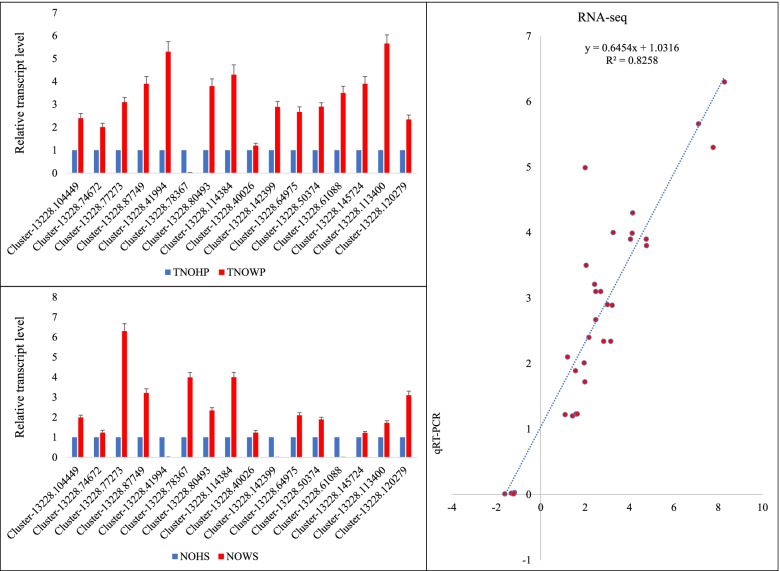
qRT-PCR analysis of the selected N. indicum genes. The bar graphs in two panels show the relative transcript level in TNOHP vs TNOWP (top) and NOHS vs NOWS (bottom). The error bars represent standard deviation. The panel of the right show correlation between RNA-seq (FPKM values) and the relative gene expression measured through qRT-PCR. Where NOWS, NOHS, NOWP, and NOHP represent diseased stem tip, healthy stem tip, diseased phloem, and healthy phloem of N. indicum

The qRT-PCR analysis was performed to validate the reliability of the RNA-seq data. For this we studied the relative gene expression of sixteen transcripts by using an *Actin-2*. Overall, the sixteen genes showed similar expression trend as of the RNA-seq for the same genes; as noted by R^2^ = 0.8258 (Fig. 8).

### Metabolome profile of *N. indicum*

**Fig. 9:**
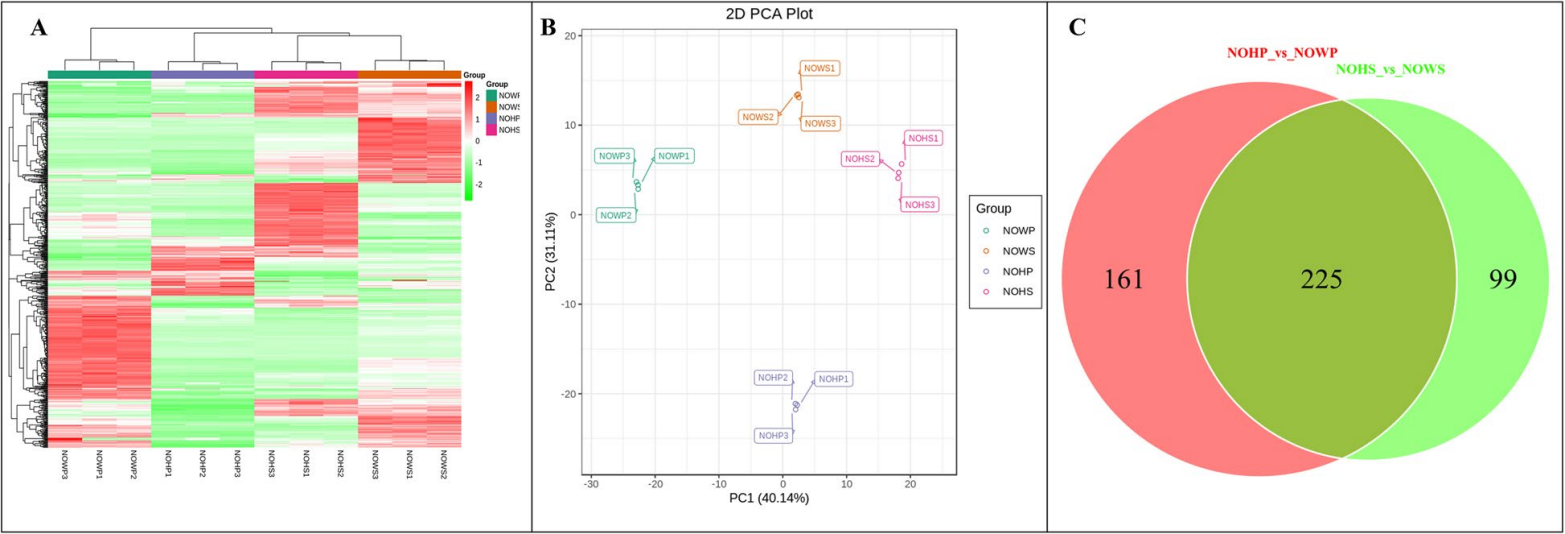
**A** Heatmap, **B** Principal component analysis, and **C** Venn diagram of the detected metabolites in N. indicum. Where NOWS, NOHS, NOWP, and NOHP represent diseased stem tip, healthy stem tip, diseased phloem, and healthy phloem of N. indicum. 1, 2, and 3 with the tissue names represent replicates

The UPLC-MS/MS analysis of the diseased and healthy *N. indicum* phloem and stem tissues resulted in the identification of 586 metabolites (Fig. 9A). The PCA showed grouping of the replicates for each treatment indicating that the sampling was reliable. PC1 and PC2 explained 40.14% and 31.11% variability, respectively (Fig. 9B).

Based on the orthogonal partial least squares-discriminant analysis (OPLS-DA) we found that 386 and 324 metabolites were differentially accumulated in NOHP vs NOWP and NOHS and NOWS, respectively (Supplementary Tables 5– 6). The OPLS-DA models showed that the Q2 for NOHP vs NOWP and NOHS vs NOWS was 0.997 and 0.996, respectively (Supplementary Fig. 5). This indicates that the OPLS-DA models are reliable. We found that 225 DAMs were common between both treatments whereas 161 and 99 DAMs were specific to NOHP vs NOWP and NOHS vs NOWS, respectively (Fig. 9C).

**Fig. 10:**
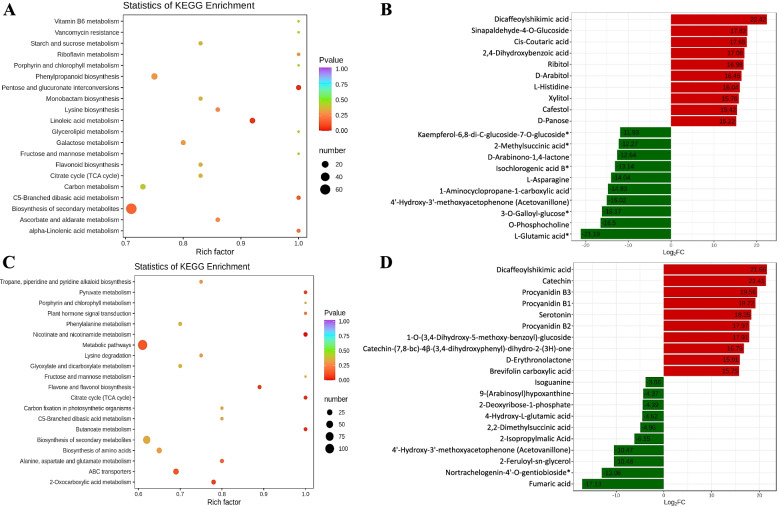
**A** Scatter plot of KEGG pathways to which DAMs were enriched in NOHP vs NOWP, **B** Top-10 (up- and down) accumulated metabolites in NOHP vs NOWP. **C** Scatter plot of KEGG pathways to which DAMs were enriched in NOHS vs NOWS, **D** Top-10 (up- and down) accumulated metabolites in NOHS vs NOWS. Where NOWS, NOHS, NOWP, and NOHP represent diseased stem tip, healthy stem tip, diseased phloem, and healthy phloem of N. indicum

The DAMs between the NOHP and NOWP were enriched in 83 different KEGG pathways. Most importantly, the DAMs were enriched in pentose and glucuronate interconversion, linoleic acid metabolism, biosynthesis of secondary metabolites, α-linoleic acid metabolism, and phenylpropanoid biosynthesis (Fig. 10A). Sixty-three and 17 DAMs were specific to NOWS and NOWP, respectively (Supplementary Table 5). Of these the top-10 metabolites that were up/down accumulated are represented in Fig. 10B. The top-10 exclusively up-accumulated metabolites were classified as phenolic acids, amino acids and derivatives, and terpenoids (Supplementary Table 5).

The DAMs between NOHS and NOWS were enriched in metabolic pathways, flavanol and flavonoid biosynthesis, pyruvate metabolism, nicotinate and nicotinamide metabolism, citrate cycle (TCA cycle), and alanine, aspartate, and glutamate metabolism (Fig. 10C). Apart from these, the DAMs were accumulated in 82 different KEGG pathways. Forty-five and four metabolites were exclusively accumulated in NOWS and NOHS, respectively. The top-10 up/down accumulated metabolites are represented in Fig. 10D. The highly accumulated (top-10) metabolites in NOWS were classified as phenolic acids, flavonoids, tannins, alkaloids, and organic acids (Supplementary Table 6). The KEGG pathway specific accumulation of metabolites is discussed below.

### Pathway specific DAM enrichment in diseased N. indicum phloem and stem

- Plant hormone signal transduction pathway
  We observed the higher accumulation of IAA and N6-isopentenyladenine in NOWP as compared to NOHP. Whereas, we observed reduced accumulation of IAA, SA, JA in NOWS as compared to NOHS (Supplementary Table 6). These observations propose the involvement of hormone signaling in *N. indicum* in the diseased tissues.
- Alanine, aspartate, and glutamate metabolism
  The differential metabolite accumulation showed that L-asparagine, L-aspartic acid, L-glutamic acid, citric acid, argininosuccinic acid, and α-ketoglutaric acid biosynthesis reduced in NOWP as compared to NOHP. These metabolites, as well as succinic acid, α-ketoglutaric acid, fumaric acid, and γ-aminobutyric acid biosynthesis decreased in NOWS as compared to NOHS (Supplementary Table 6). Overall, these observations suggest that the witches’ broom infection caused reduction in these metabolites as compared to respective control.
- Linoleic acid and α-linoleic acid metabolism
  We observed the accumulation of metabolites associated with the α-linoleic acid metabolism in NOWP as compared to NOHP. In case of NOWS vs NOHS, only one metabolite (9-Hydroxy-12-oxo-15(Z)-octadecenoic acid) was differentially accumulated. Similarly, all the metabolites that were enriched in linoleic acid metabolism were up-accumulated in NOWP as compared to NOHP. Whereas, the number of metabolites differentially accumulated between NOHS and NOWP was lower but a similar accumulation trend was observed as in case of phloem. These results are consistent with the transcriptome findings (Supplementary Table 4; Fig. 6).
- Phenylpropanoid biosynthesis and flavonoid biosynthesis pathway
  We observed higher accumulation of cinnamic acid, p-coumaric acid, caffeic acid, ferulic acid, p-coumaroylshikimic acid, coniferyl-aldehyde, coniferyl-alcohol, and p-coniferyl alcohol in diseased phloem tissues as compared to control. The increased accumulation of these metabolites probably leads to the higher biosynthesis of coniferin (Supplementary Table 6). These observations are consistent with the observations of enrichment of DEGs in the same pathway (Supplementary Table 4), thus confirming that coniferin biosynthesis is increased in response to the infection. Similarly, the accumulation of most of the metabolites enriched in this pathway were up-accumulated in NOWS as compared to NOHS (Supplementary Table 6). These observations propose that increased biosynthesis of phenolic acids is a generalized response (regardless of infection site) of *N. indicum* plants against the infection.
  The accumulation of flavonoids and phenolic acids that are biosynthesized in flavonoid biosynthesis pathway was increased in NOWP and NOWS as compared to their respective controls (Table 2). Similar observations were recorded in case of transcriptome sequencing (Supplementary Table 4).
  Taken together, it could be proposed that large scale flavonoid and phenolic acid biosynthesis occurs in the diseased *N. indicum* plants.
- Citrate cycle and nicotinate and nicotinamide metabolism

Five and six metabolites were differentially accumulated in citrate cycle in NOWP vs NOHP and NOWS vs NOHS, respectively. Among these, succinic acid was up-accumulated in NOWP as compared to NOHP, whereas the accumulation of all other metabolites was decreased in response to infection. Similarly, all the metabolites (except phosphoenolpyruvate) were down-accumulated in NOWS as compared to NOHS (Supplementary Table 6). These observations indicate that Withes’ broom infection greatly influences citrate cycle.

The reduced accumulation of nicotinate, nicotinate D-ribonucleoside, succinate, and 4-amino butanoate was observed in NOWP as compared to NOHP (Supplementary Table 6). On the other hand, an increased accumulation of β-Nicotinamide mononucleotide, succinic acid, nicotinamide, nicotinic acid, nicotinic acid adenine dinucleotide was observed in NOWP and/or NOWS as compared to the respective controls. These metabolites are present upstream the tryptophan metabolism, alanine, aspartate and glutamate metabolism, tropane, piperidine, and pyridine alkaloid biosynthesis, alanine metabolism, and citrate cycle. Thus, their differential accumulation in response to the witches’ broom infection possibly effects all the downstream pathways.

## Discussion

### Dominant pathogen in witches’ broom diseased N. indicum

We conducted a survey of the Guangdong province in China to determine the disease incidence of WBD on *N. indicum* natural plantations. The results that WBD was prevalent in rural areas as compared to urban ones can be linked with the lack of proper management practices. Since we found that this disease can impact > 80% plantations, thus, we performed detailed experiments to study possible casual organism and what kind of defense responses are activated by *N. indicum* plants. We performed PCR, nested PCR, and transmission electron microscopy analyses and found that the disease is not associated with phytoplasmas as in the case of Jujube madness disease [43], Paulownia arbuscular disease [9], and [44] (Fig. 2). Thus, these analyses remove the possibility that the causal agent of the *N. indicum* WBD is phytoplasma. Thus, the results of ITS sequencing using MiSeq documents that the possible pathogen causing WBD in *N. indicum* is *Cophinforma mamane* (Fig. 3)*.* Though limited knowledge is available on the *Botryosphaeria mamane* (a homotypic synonym of *C. mamane*), but this fungus has not been restricted to *S. chrysophylla* since it has also been reported on *Acacia mangium* and *Eucalyptus urophylla* in Venenzuela. These reports were based on ITS phylogeny as well as on the basis of similarity of the conidial characteristics. Mohali*, *et al*.* [45]. Zhou and Stanosz [46] sequenced the ITS regions of these two strains of *B. mamane* (from *A. mangium* and *E. urophylla*) however, the ITS phylogeny reported by Naito, Tanaka, Taba, Toyosato, Oshiro, Takaesu, Hokama, Usugi and Kawano [44] has been debated [47]. A global survey on the ecology and diversity of the endophytic fungi indicated the presence of *C. mamane* in different plant species such as *Garcinia mangostana*, *Bixa Orellana*, and *Catharanthus roseus* [48], indicates that this fungi can use different plant species as host and infect them. An earlier study had showed that *B. mamane* was found associated with WBD symptoms such as branch contortions and swellings, leading to a death of the tissues in *Sophora chrysophylla* [49]. This is very similar to the findings of our survey (Table 1). Other than *S. chrysophylla,* this fungal species has also been reported to be associated with the decline of the table grapevine in North-eastern Brazil [50]. Thus, our data is in accordance with these reports and the infection in *N. indicum* plants is potentially due to *C. mamane.*

There are several strategies on the disease control (including WBD) such as use of genetic resistance (genetic enhancement), cultural management such as removal of the diseased branches, agroforestry production systems, chemical control (fungicides), biological control (microbiological fungicide), and integrated pest management [51, 52]. Our survey results indicate that the diseased areas in Guangdong should be treated in order to avoid the spreading of the disease to the whole province. Since we found a lower disease incidence in urban areas and parks, therefore, it is understandable that the disease can be managed by following regular pruning and trimming of the diseased branches. Particularly, the areas with > 80% disease incidence should be a priority to remove or prune the diseased trees since the removal of the diseased branches is the cheapest and the most effective practice. For the areas, where the disease incidence is lower e.g., urban areas with < 5% disease index, attention should be given to maintain tree vigor and enhance disease resistance. A better strategy could be planting multi-variety model. So that plants with variable resistance can grow. In this regard, the detailed understanding of the infection and *N. indicum* responses could be helpful to devise a suitable control strategy. The key pathways that were regulated in the diseased *N. indicum* plants are discussed below.

### MAPK cascade is possibly involved in responses against witches’ broom disease in N. indicum

Our results displayed the activation of MAPK-signaling plant pathway in diseased phloem and stem (Figs. 2–3; Supplementary Table 4). MAPK cascades are highly conserved signaling networks in plants and play an important role in signaling when a plant is under pathogen attack [53]. Particularly, pathogen/microbe-associated molecular patterns (PAMPs/MAMPs) results in the activation of mitogen activated protein kinases (MAPKs) [54]. The increased expression of BAK1, MKK1/2, MKS1, MPK, MPK3/6, ACS6, WRKY TF, PR1, and FRK1 (Fig. 4) in NOWP and/or NOWS proposes that *N. indicum* responds to witches’ broom infection by both the early and late defense responses. This proposition is based on the known roles of genes involved in MAPK signaling cascade e.g., most recently, three genes i.e., MKK2, MPK2, and MKK4 were reported to be involved in responses against WBD in Chinese Jujube [55]. It was established in Arabidopsis that constitutive expression of MKK2 caused the increased the expression genes encoding enzymes for the biosynthesis of JA and ethylene [56]. Since these expression changes correspond to that of JA and ethylene related transcripts (Fig. 7). Therefore, the defense response in *N. indicum* involves both phytohormone biosynthesis and MAPK-signaling. Similarly, a tomato MKK4 (together with MKK2) have been reported to be induced by *Botrytis cinerea* and JA and ethylene [57]. Whereas its silencing reduced resistance in tomato plants against *B. cinerea*. Thus, the expression changes in the diseased and healthy *N. indicum* plants correspond to these observations and indicate that *N. indicum* plants have similar defense strategy. However, detailed characterization must be carried out to confirm the individual/combined role(s) of these genes. Another defense strategy that *N. indicum* plants might adapt as a part of MAPK signaling cascade is the production of H_2_O_2_, which is known for a central role in signaling pathways within and between plant cells, especially when under pathogen attack [58]. This is probably due to the increased expression of OXI1, ANP1, NDPK2, MPK3/6, and WRKY TFs in NOWP and/or NOWS as compared to their respective controls (Fig. 4; Supplementary Table 4). Since these genes are involved in programmed cell death (PCD), therefore, there is a possibility that the oxidative burst (due to the accumulation of H_2_O_2_) is activating PCD in diseased *N. indicum* plant tissues [59]. This proposition is further supported by the changes in the expression of genes such as MKK3, RobhD, and OXI1 in NOWP and NOWS, as compared to their controls. MKK3 and CaM4 are present upstream the OXI1 [60]. Taken together, our results indicate that *N. indicum* plants activate MAPK signaling cascades which result in early and late defense responses against pathogen, H_2_O_2_ production, cell death, and maintenance of homeostasis of ROS.

### N. indicum may use plant-hormone signal transduction pathway during WBD

The results that both transcriptome and metabolome analysis showed the enrichment of DEGs and DAMs in plant-hormone signal transduction pathway, indicates during the witches’ broom infection, phytohormone signaling might play important roles (Fig. 5; Supplementary Table 4). Earlier studies have shown that plant hormones such as SA, JA, and ethylene act as signal molecules and can trigger a range of defense responses [61]. The upregulation of NPR, TGA, and PR1 in NOWS and/or NOWP as compared to the controls indicate that SA signaling is activated in *N. indicum* plants when diseased (Fig. 4; Supplementary Table 4). This is consistent with the metabolome findings where we observed the changes in SA in NOWS (Supplementary Table 6). Silencing of NPR genes (NPR1 and NPR3) in *C. roseus* altered the susceptibility against Periwinkle leaf yellowing [62]. Thus, it could be proposed that WBD suffering *N. indicum* plants use SA-induced defense responses to activate PR1, which is a known for its role in resistance against invading pathogens; most recently the upregulation of PR1 was reported in cacao against WBD [63]. Apart from SA, JA signaling might also be a possible defense strategy in *N. indicum* plants against WBD. This proposition is based on the observations that genes and metabolites associated with JA signaling were differentially regulated/accumulated in the studied tissues (Fig. 5 & 7; Table 3; Supplementary Table 4). Also, this is consistent with the findings that large scale JA changes occur in Chinese jujube leaves showing WBD symptoms due to phytoplasma infection [64]. These expression changes further confirm the above discussed roles of MKK2 and MKK4 genes in tomato and Arabidopsis [56, 57]. Similar defense strategy has been reported in *Paulownia fortunei* to paulownia witches’ broom infection [65]. Apart from SA and JA signaling, the observation that a large number of transcripts annotated in ethylene signaling pathway indicates the probable role of ethylene in *N. indicum* against witches’ broom infection. The ETR and CTR1 genes initiate MAPK signaling cascade, which is consistent with the differential expression of the MKK transcripts in the diseased and healthy *N. indicum* tissues [66]. Their higher expression, along with SIMKK, EBF1A/2, and ERF1/2 in NOWS and/or NOWP as compared to controls indicate that *N. indicum* plants initiate MAPK signaling cascade as a response to WBD. These results are consistent with the observation of the increased levels of ethylene in cocoa shoots after witches’ broom infection [12]. The observation that most genes in the brassinosteroid signaling pathway were downregulated in NOWS, while upregulated in NOWP as compared to their respective controls indicates that WBD may lead towards reduced cell division and elongation in stem, but an increased cellular growth and division in phloem. These observations are consistent with the disease morphology that internodes are shortened and sprouting of the auxiliary buds [67]. Thus, overall regulation of the plant-hormone signaling pathway in WBD suffering *N. indicum* signifies the roles of respective hormone signaling networks in resistance and responses to WBD. Particularly, these observations highlight that JA, SA, and ethylene biosynthesis and signaling related genes interact with the key genes in MAPK signaling pathway and help plant to withstand the disease.

### Plant-pathogen interaction pathway is active in WBD suffering N. indicum plants

Plants respond to the invading pathogens through multiple layers of specific immunity mechanisms [68]. When pathogens invade plants, the cytosolic Ca^2+^ concentrations change rapidly and is considered an essential early event during disease infection in plants [69]. The observation that Robh gene was upregulated in NOWP and/or NOWS as compared to controls indicate the possibility of changes in Ca^2+^ levels in the diseased tissues since both Rboh and CDPK genes are regulated by Ca^2+^. This upregulation leads to increased ROS accumulation, which eventually triggers defense reactions including cell wall reinforcement [70]. The upregulation of defense related genes i.e., WRKY TFs [71], FRK1 [72], NHO1, and PR1 [73] as a result of the changes in the expression of MAPK signaling cascade related genes indicates a strong defense response to WBD [71–73] [74]. It is interesting to note that the observed defense responses in *N. indicum* are similar to cacao and jujube [75]. The expression of genes such as MKK1/2, PTIs, and RAR1 is another signal apart from possible changes in Ca^2+^ concentrations. Previously, it is known that PTI5/6 regulates defense responses and disease resistance e.g., in tomato against *Stemphylium lycopersici* [76, 77], while RAR1 is specifically required for plant innate immunity [72, 76, 77]. Thus, it could be stated that *N. indicum* plants’ defense mechanism against witches’ broom infection include possible changes in Ca^2+^ concentrations in cytosol, which together with MAPK-signaling cascade, activates related defense pathways. However, these observations are a preliminary picture of the *N. indicum*-*C. mamane* interaction, and future studies should focus on the identification of resistant and tolerant *N. indicum* genotypes to WBD followed by the identification of perception of *C. mamane* by host plants.

### Phenylpropanoid and flavonoid biosynthesis is increased in diseased N. indicum stem and phloem

Phenylpropanoid compounds play a range of defense related functions in plants such as preformed/inducible physical and chemical barriers. These compounds are also involved in local or systemic signaling and may induce defense related genes [78]. The enrichment of DEGs and DAMs in phenylpropanoid biosynthesis pathway is consistent with the earlier reports in *Paulownia fortunei* [65], cacao [63, 74], and green tea [65]. Particularly, the upregulation of trans-cinnamate 4-monooxygenase, CCOAOMT1, 4CL, Kat, CCR, F5H, PAL, CSE, and CAD in NOWP and/or NOWS as compared to respective controls possibly lead the increased accumulation of lignans and coumarins and phenolic acids (Supplementary Table 6). Earlier studies have demonstrated that higher expression of CCOAOMTs and CADs lead to higher lignin biosynthesis and disease resistance in *Arabidopsis*, respectively [79–81]. Similarly, it is known that F5H plays role in phenylpropanoid biosynthesis in *Arabidopsis* [82], while the activity of PAL increases in Mexican lime plants showing WBD symptoms [15]. Thus, it could be proposed that the witches’ broom infection in *N. indicum* leads towards the increased expression of the above-mentioned genes, which in turn causes the accumulation of phenolic acids and lignans and coumarins.

Since flavonoid biosynthesis pathway is present downstream the phenylpropanoid biosynthesis [83], therefore, the accumulation of flavonoids and phenolic acids could be expected. This higher accumulation of flavonoid and phenolic acids could be related with the increased expression of naringenin 3-dioxygenase (F3H), trans-cinnamate 4-monooxygenase (C4H), CCOAOMT, chalcone synthase (CS), chalcone isomerase (CHI), flavonol synthase (FLS), and other genes enriched in this pathway (Supplementary Table 4). The study on Mexican lime tree suffering from WBD showed the upregulation of these genes as compared to controls [6]. Thus, it could be concluded that increased flavonoid biosynthesis is a common response of different plants species to the pathogen infection [78, 84] and that the *N. indicum* plants manipulate flavonoid biosynthesis together with phenylpropanoid biosynthesis pathway to resist against this disease.

### Linolenic acid and α-linolenic acid metabolism may be a part of defense responses against WBD

As we know that linoleic acid is a precursor of JA, which is involved in defense responses in plants [85] and references therein]. The end product of α-linoleic acid metabolism is Jasmonate/methyl-Jasmonate, which induces defense responses in plants against different biotic stresses [86]. In this regard, the regulation of multiple genes controlling latter stages of Jasmonate/methyl-Jasmonate biosynthesis related transcripts i.e., COR, acetyl-CoA acyltransferase 1, phospholipase A1 in NOWP and/or NOHP. suggests that *N. indicum* plants use similar mechanism for defense against WBD (Supplementary Table 4; Fig. 5). These changes in the expression are consistent with the observations that metabolites associated with the α-linoleic acid metabolism in NOWP and NOWS as compared to their respective controls (Supplementary Table 6). The changes suggest that *N. indicum* plants adapt a strategy to use JA induced defense responses against the pathogen infection regardless of the site of infection i.e., stem or phloem.

### Possible roles of other pathways in defense responses in N. indicum against WBD

The differential regulation of other pathways such as citrate cycle, nicotinate and nicotinamide metabolism, and alanine, aspartate, and glutamate metabolism indicate that *N. indicum* plants adapt a multi-layer response to the witches’ broom infection. Previous studies have shown that the flux of tricarboxylic acid (TCA) may play role during the setup of plant defenses mainly because it is a central pathway for the generation of primary metabolites in order to recruit and redistribute energy flows [87, 88].

The differential accumulation of compounds enriched in nicotinate and nicotinamide metabolism pathway such as β-Nicotinamide mononucleotide, succinic acid, nicotinamide, nicotinic acid, nicotinic acid adenine dinucleotide is consistent with the changed expression of the genes enriched in this pathway (Supplementary Tables 2–3). These compounds are precursors of nicotinamide adenine dinucleotide (NAD) [89]. The expression of genes associated to the above-mentioned metabolites in this pathway have been previously shown to increase in response to disease infection [90], which is consistent with our findings. It has also been reported that extracellular pyridine nucleotides induce PR genes in *Arabidopsis* [91]. Since we have seen the upregulation of PR gene in NOWS and NOWP, therefore, it is possible that a similar response exists in *N. indicum* with WBD. Furthermore, because nicotinate and nicotinamide metabolism are present upstream the alanine, aspartate, and glutamate metabolism, and tryptophan metabolism [92], therefore, the differential accumulation of metabolites related to these pathways is understandable. At this point, it could be proposed that under WBD attack, *N. indicum* might stimulate the variable accumulation of metabolites related to alanine, aspartate, and glutamate metabolism. However, a detailed understanding of how these pathways might regulate the defense responses is needed through gene/pathway-specific investigations.

## Conclusions

The survey of Guangdong province, China showed that the WBD is widespread in the province and the disease incidence can be up to 80%. Considering this alarming situation, we used MiSeq based ITS sequencing and found that *C. mamane* was the most prevalent microbe in the diseased tissues. We also confirmed the absence of phytoplasma in the diseased *N. indicum* tissues. Further, our combined transcriptome sequencing and metabolome profiling of WBD suffering *N. indicum* phloem and stem indicated the multi-layer defense responses in this plant species. Particularly, we found that plant-pathogen interaction, plant-hormone signal transduction, MAPK-signaling (plant), phenylpropanoid biosynthesis, flavonoid biosynthesis, linoleic acid metabolism, α-linoleic acid metabolism, nicotinate and nicotinamide metabolism, and alanine, aspartate, and glutamate metabolism pathways are activated during the WBD in phloem and stem. WBD in *N. indicum* triggers PAMP and other defense related signals such as MAPK signaling cascade, which possibly result in early and late defense responses against pathogen, H_2_O_2_ production, cell death, and maintenance of homeostasis of ROS. This study presents a wide range of target genes belonging to the above-mentioned pathways for gene specific characterization and developing WBD resistant *N. indicum* plants by using CRISPR/Cas and other gene manipulation techniques [93]. Besides, our sampling will allow us in a future study a detailed characterization of the pathogen conferring the WBD in *N. indicum*.

## Methods

### Survey of diseased areas

The current study was based on *Nerium indicum* plants from Guangdong province in China. The samples were obtained from the wild and no permissions were necessary to collect such samples. Also, the collection of plant material complies with Chinese Academy of Forestry’s, Chinese, and international guidelines and legislation. The formal identification of the samples was undertaken by the corresponding author of this publication (Professor Haibin Ma). The voucher specimens have been deposited in a local herbarium of Research Institute of Tropical Forestry, Chinese Academy of Forestry (Guangzhou, China), under the ID: NRX001PR20023.

We conducted a survey of ten different cities of Guangdong province, China i.e., Dongguan, Gaozhou, Guangzhou, Huizhou, Jieyang, Meizhou, Shenzhen, Zhongshan, Zhuhai, and Shaoguan and recorded the disease incidence (see Table 1 for the number of samples studied from each area). The samples were considered diseased if the plants/branches showed typical WBD symptoms. The disease incidence and index were determined according to the following formulas.

$$\mathrm{Disease incidence rate}=\frac{\mathrm{Numbers of onset}}{\mathrm{ the total number of Investigated plants}}\times 100$$

$$\mathrm{Disease index}=\frac{\Sigma (\mathrm{Disease stage number}\times \mathrm{On behalf of the numerical})}{\mathrm{the total number of Investigated plants}\times \mathrm{The highest disease}-\mathrm{grade representative value}}\times 100$$

The disease severity was judged on the basis of following scale (Table 5).

**Table 5:** Disease severity scale of witches’ broom disease in *N. indicum*

Infection category	Grading of witches’ broom disease at individual level	Grading of witches’ broom disease at branch level	Value/Score
Level 1	No observable infection	No observable infection	0
Level 2	The % of infected branches to the total was < 25%	The twig grows in an acropetal sequence with very thin stems and little leaves, but only for single twig without cluster phenomenon	1
Level 3	The % of infected branches to the total was 26–50%	The lateral buds grew out, several lateral branches clustered together, and the smaller leaves were not withering	2
Level 4	The % of infected branches to the total was 51–75%	The lateral buds repeatedly grew out with distributed small clumps, and the smaller leaves either withered or fell off	3
Level 5	The % of infected branches to the total was > 75%	The lateral buds repeatedly grew out with comprehensively distributed clumps of the culm	4

Authors identified *Nerium indicum* cv. Plenum (Reddish flowered plants) plantations severely showing WBD symptoms in Shaoguan, China (114.1 longitude and 24.5 latitude). The soil type of the sampling location is yellow–red. At the time of sampling, the plants were 8–10 years old. The average temperature of the area is 26 ℃.

### Disease identification and pathogen confirmation

**Fig. 11:**
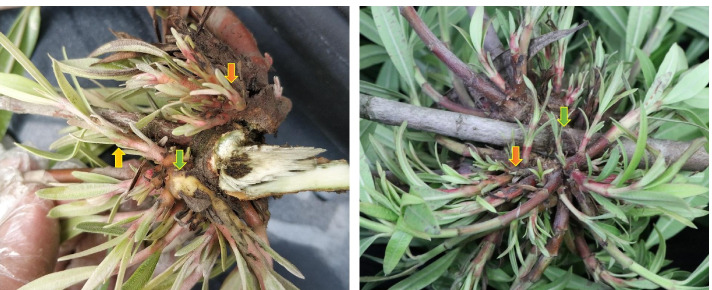
Plant samples showing the witches’ broom disease symptoms. Dark orange arrows = germination of a large number of axillary buds and lateral branches, green arrows = swollen base, and dark yellow arrow = shortening of internodes

The occurrence of arbuscular symptoms was used as the basis for judging the infection. The growth of the main shoot is stagnant, axillary buds or a large number of side branches germinate followed by the shortening of the arbuscular branches between nodes. Also, the swollen tissue appeared at the base of the sprouting witches. The base of the newly grown lateral branches appears swollen, reddish, and leaves become smaller and yellower. The branches show reduced flowering, while the disease worsens year by year. The branches had tumors, the cortex was decomposed, and in a state of ulceration. Some branches were crumpled and curved. Upon cutting open the diseased branches, blackened fibrous bundles were observed. Triplicate Nerium plants suffering from WBD were sampled. Stem tip (NOWS) and phloem (NOWP) of the diseased plants were taken by peeling the stem with scalpel, rinsed thrice with sterile water, and stored in liquid nitrogen for pathogen identification, RNA extraction, and metabolite analyses. Three healthy Nerium plants were used to harvest stem tip (NOHS) and phloem (NOHP) to be used as control (Fig. 11).

The NOWP tissues were dissected, a 5 mm × 5 mm black rotten xylem tissue was cut (in ultra-clean work bench), immersed in 70% ETOH for 1 min, and sterilized in 3% hydrogen peroxide for 20 s. The tissue was then washed thrice with sterile water, dried on a filter paper, and placed on a potato dextrose agar (PDA) plates (four tissue pieces per plate), and incubated for 3–4 days. The hyphae growing at both ends were transferred to a fresh PDA plate. The fungal hyphae were isolated, and used for DNA extraction.

After surface sterilization, the DNA was extracted from 1 g of each sample by following the modified method of Griffiths*, *et al*.* [94] as reported by Monard*, *et al*.* [95]. The quality of DNA was checked on 1% agarose gel electrophoresis. The PCR amplifications were carried out by using a barcode specific primer i.e., ITS1F and ITS2R as reported earlier [96, 97]. The reactions were carried out on an ABI GeneAmp® 9700. The PCR reaction conditions were as reported earlier [95]. Each sample was amplified thrice, the triplicate products were pooled, loaded on 2% agarose gel electrophoresis, and recovered by using AxyPrep DNA Gel Extraction Kit (Axygen, USA). The eluted sample is detected and quantified by suing QuantiFluorTM-STBlue fluorescence quantitative system (Promega, USA). The DNA was then prepared by using TruSeqTM DNA Sample Prep Kit to be sequenced with MiSeq.

The sequencing reads were filtered in Flash (version 1.2.11) [98] and only quality reads were used for further bioinformatic analyses using standard microbial analysis pipeline. We performed an OUT-cluster analysis at 97% identity using UPARSE (version 7.0.1090) [99], and then globally aligned using USEARCH [100] against a database of high quality ITS fungal gene sequences UNITE (https://unite.ut.ee/), Silva (Release138 http://www.arb-silva.de), RDP (Release 11.5 http://rdp.cme.msu.edu/), Greengene (Release 135 http://greengenes.secondgenome.com/), and Functional gene FGR (Release7.3 http://fungene.cme.msu.edu/) to determine taxonomic classifications by RDP Classifier [101]. A phylogenetic tree was constructed in IQ-TREE [102] after alignment in MAFFT [103].

To test if the disease was caused by phytoplasma or fungus, we performed PCR and nested PCR reactions on the genomic DNA extracted from the diseased Jujube, Paulownia, and *N. indicum* samples. The genomic DNA was extracted as reported above. The 16S rRNA gene was amplified using genomic DNA as a template by employing the common primers P1/P7 and R16mF2/R16mR1 of the phytoplasma. PCR was carried out in a 30 µL reaction system; 1 µL DNA, 0.75 µl of forward and reverse primer, 2 × PCR master mix (0.05 U·μL^−1^ Taq DNA polymerase, 4 mM MgCl_2_ and 0.4 mM dNTPs), and ddH_2_O. The reactions were carried out for 35 cycles with the conditions were as follows. 94 °C for 5 min, 94 °C for 30 s, 53 °C for 40 s, 72 °C for 2 min, followed by a final extension for 10 min at 72 °C. Products for both PCR types were detected on 1% agarose gel electrophoresis. The transmission electron microscopy was done as reported earlier by Park*, *et al*.* [104].

### Transcriptome analyses

#### RNA extraction, library preparation, and sequencing

High-quality RNA was extracted from the triplicate samples of phloem and stem tips of both diseased and healthy plants. RNA was extracted using Spin Column Plant total RNA Purification Kit (Sangon Biotech, Shanghai, China). The quality of the RNA was tested by analyzing the integrity (using agarose gel electrophoresis and Agilent 2100 bioanalyzer) and concentration (by Qubit 2.0 Fluorometer).

To prepare libraries, mRNA was purified from total RNA by using poly-T oligo-attached magnetic beads followed by breaking it into short RNA fragments by using fragmentation buffer. The short-fragments were then used to synthesize first strand cDNA with random hexamers, buffer, and dNTPs, DNA polymerase I. The double-stranded cDNA was purified by using AMPure XP beads. The purified cDNA was repaired, A-tailed, and ligated with a sequencing adapter and then AMPure XP beads were used for fragment size selection, and finally PCR enrichment was performed to obtain a final cDNA library. Once the libraries were prepared, their quality was tested preliminarily by Qubit 2.0 and Agilent 2100 for insert size detection. This was followed by Q-PCR to determine the effective library concentration (> 2 nM). The libraries were then sequenced on Illumina HiSeq platform (Illumina Inc., San Diego, CA, USA).

#### Bioinformatic analyses

Raw Illumina HiSeq sequencing data was processed for quality control by removing reads with adaptors, removing paired reads if N content in sequencing reads exceeded 10%, and if low quality basis (Q ≤ 20) in sequencing reads exceeded 50%. This was followed by the determination of error distribution and GC content in the sequencing reads.

BLAST [105] was used to compare unigene sequences with KEGG [106], NR [107], Swiss-Prot [108], GO [109], COG/KOG [110], Trembl databases [111]. Furthermore, we predicted unigenes’ amino acid sequences and used HMMER software to compare the sequences with Pfam [112].

To quantify the gene expression, the spliced transcripts by Trinity were used as reference sequence and the clean reads of each sample were mapped to it by using bowtie2 [113] in RSEM [114]. We then calculated the Fragments Per Kilobase of transcript per Million fragments mapped (FPKM) as an index of the expression level of the transcripts. Overall FPKM values were visualized as box-plot in R. The expression data was then used to study the Pearson’s Correlation Coefficient (PCC) and Principal Component Analysis (PCA) in R. Further we used DESeq2 [115] to find the differentially expressed genes (DEGs) between the diseased and healthy samples. Then Benjamini–Hochberg method [116] was used to perform hypothesis test correction on p-value to obtain false discovery rate (FDR) and screened the DEGs on the basis of FDR (< 0.05) and log2 foldchange (≥ 1). Venn diagrams were prepared in InteractiVenn [117].

KEGG pathway enrichment analysis of the DEGs was done in KOBAS2.0 [118] FDR (< 0.05) was used to reduce false positive prediction of enriched KEGG pathways. The degree of KEGG enrichment was measure by Rich factor, Q-value, and the number of gene enriched in each pathway and was displayed as scatter plots (20 entries maximum).

Finally, we used iTAK software to predict plant transcription factors (TF); it integrates PlnTFDB and PlantTFDB and uses TF family and identifies TF through HMM-HMM scan comparison [119].

#### Quantitative real-time PCR analysis

We selected sixteen genes from the *N. indicum* RNA-seq comparison data to validate the sequencing results. The *Actin-2* gene was used as an internal control. The PCR reactions and determination of the relative gene expression were carried out as reported earlier [120] on a Rotor-Gene 6000 machine (Qiagen, Shanghai, China) using primers designed in Primer 3 (http://frodo.wi.mit.edu/primer3/) (Table 6). The thermal cycling profile was as follows. 50 °C for 2 min and 95 °C for 2 min, followed by 40 cycles at 95 °C for 3 s and 60 °C for 30 s. We also performed the melting curve analysis and verified the single product amplification with temperature ranging from 55 to 95 °C by increasing of 1 °C every step. Volume for all the reactions was 10 μL; 30 ng of cDNA, 5 μL 1 × SYBR® Select Master Mix (Applied Biosystem, Carlsbad, CA, USA), and 0.2 μL (20 μM) of each primer. Three biological replicates were analyzed in independent runs.

**Table 6:** List of forward and reverse primers that were used for the quantitative real-time PCR analysis of the *N. indicum* genes

Gene ID	Reverse primer sequence	Forward primer sequence
*Cluster-13228.104449*	GAACGTTGCCTGATTTT	CATCACACTACATTTC
*Cluster-13228.74672*	ACATTCTGGCTTTGGC	CAGGGTTTATGGAGGG
*Cluster-13228.77273*	GTACTTTCTTTCATACT	TCTATGACTTGGAGG
*Cluster-13228.87749*	CGGTTCCATTCTTCTT	TTCTTGAATCGGGAGG
*Cluster-13228.41994*	GAACGCTTTCTCCTCA	TCGTCCAGTCTCCA
*Cluster-13228.78367*	CAAGGGCTAAGGTTCA	TATCCACGCATGGCAG
*Cluster-13228.80493*	GCTGTCCGTGTCAATC	CCATTGCACTGGCGAA
*Cluster-13228.114384*	GCCTAAAGGTCCTCTGT	AGTGTTGGCTTTGTCTAT
*Cluster-13228.40026*	GCCCGTTCACCAAGTC	CTACAAATGGAACAAG
*Cluster-13228.142399*	GGTACAATCTCCATC	GTGCGGCAGAAGTGTC
*Cluster-13228.64975*	TCCCTGTTTCCACCTC	GTTACCACGGACACGA
*Cluster-13228.50374*	TTATTTCCTCGGATTCTC	TGCTGGAACTACTGACG
*Cluster-13228.61088*	ATGCGTTCACCACACTC	TGATGCCTCAGTGTTG
*Cluster-13228.145724*	ATCCCTTGGCAGTAAA	ATTTAGGCCTAGTAT
*Cluster-13228.113400*	AAGCCTTAGAGCCAA	ATTCGTAAACCCTCAG
*Cluster-13228.120279*	GTAGTCCCATATGTTCT	TCAATTAGAATTGACCTT
*Actin-2*	AATTACGACGAGCA	CAAGCATTGGGATTTT

### Metabolome profiling

#### Sample preparation and extraction

The freeze-dried samples were crushed into powder with zirconia bead for 1.5 min at 30 Hz in a MM400-Retsch mixer mill. 100 mg powder was then extracted at 4 ℃ for 12 h in 70% MeOH (0.6 mL) followed by centrifugation for 10 min at 10,000 g. the extracts were absorbed (CNWBOND Carbon-GCB SPE Cartridge, 250 mg, 3 ml; ANPEL, Shanghai, China) and filtered (0.22 µm) prior to UPLC-MS/MS analyses.

#### UPLC conditions

To analyze the extracts, we used UPLC-ESI–MS/MS system (UPLC, Shimpack UFLC SHIMADZU CBM30A system. MS, Applied Biosystems 4500 Q TRAP). We used an Agilent SB-C18 UPLC column for the analysis. The mobile phase included solvent A, pure water + 0.1% formic acid, solvent B, acetonitrile. The measurements were recorded by setting up a gradient program that employed the starting conditions of 95% A, 5% B. Within 9 min, a linear gradient to 5% A, 95% B was programmed, and a composition of 5% A, 95% B was kept for 1 min. subsequently, a composition of 95% A, 5.0% B was adjusted within 1.10 min and kept for 2.9 min. During the measurements, the temperature for the column oven was kept 40 ℃. An injection volume of 4 µL was used. The effluent was alternatively connected to an ESI-triple quadrupole-linear ion trap (QTRAP)-MS.

#### ESI-Q TRAP-MS/MS

The ESI-Q TRAP-MS/MS settings were as reported earlier [121]. Briefly, LIT and triple quadrupole (QQQ) scans were acquired on a triple quadrupole-linear ion trap mass spectrometer (Q TRAP), API 4500 Q TRAP UPLC/MS/MS System, equipped with an ESI Turbo Ion-Spray interface, operating in positive and negative ion mode and controlled by Analyst 1.6.3 software (AB Sciex). The ESI source operation parameters were as follows: ion source, turbo spray; source temperature 550 ℃; ion spray voltage (IS) 5500 V (positive ion mode)/ -4500 V (negative ion mode); ion source gas I (GSI), gas II (GSII), curtain gas (CUR) were set at 50, 60, and 30.0 psi, respectively; the collision gas (CAD) was high. Instrument tuning and mass calibration were performed with 10 and 100 μmol/L polypropylene glycol solutions in QQQ and LIT modes, respectively. QQQ scans were acquired as MRM experiments with collision gas (nitrogen) set to 5 psi. Declustering potential (DP) and collision energy (CE) for individual MRM transitions was done with further DP and CE optimization. A specific set of MRM transitions were monitored for each period according to the metabolites eluted within this period.

#### Metabolite data analyses

Unsupervised PCA was performed in R using prcomp function. The original data was compressed into n principal components (PC1 and PC2) to describe the characteristics of the original data set. Hierarchical cluster analysis and PCC between the samples was computed and represented as heatmaps in R using pheatmap and cor functions.

To determine if the detected metabolite was differentially accumulated, we used variable importance in projection (VIP) ≥ 1 and log2 foldchange ≥ 1 as criteria. The VIP values were extracted from OPLS-DA result, which was generated using R package MetaboAnalystR; the data was log transformed and mean centered before OPLS-DA. We also performed a permutation test (200 permutations) to avoid overfitting.

The metabolites were annotated using KEGG Compound database (http://www.kegg.jp/kegg/compound/) and then mapped to KEGG Pathway database (http://www.kegg.jp/kegg/pathway.html). Pathways with significantly regulated metabolites mapped to were then fed into MSEA (metabolite sets enrichment analysis), their significance was determined by hypergeometric test's *p*-values.

## Supplementary Information

**Additional file 1: Supplementary Table 1. **Summary of transcriptome sequencing of infected and non-infected Nerium indicum L. tissues.** Supplementary Table 2. **Differential expression of genes between infected (NOWP) and non-infected (NOHP) N. indicum phloem.** Supplementary Table 3. **Differential expression of genes between infected (NOWS) and non-infected (NOHS) N. indicum stem.** Supplementary Table 4.** List of differentially expressed genes that were enriched in specific pathways in WBD infected N. indicum phloem (NOWP) and stem tip (NOWS) as compared to controls i.e., NOHP and NOHS, respectively.** Supplementary Table 5. **Details of the differentially accumulated metabolites between infected (NOWP) and non-infected (NOHP) N. indicum phloem.** Supplementary Table 6. **Details of the differentially accumulated metabolites between infected (NOWS) and non-infected (NOHS) N. indicum stem tip.** Supplementary Table 7. **Pathway specific differential accumulation of metabolites in infected N. indicum phloem and stem as compared to non-infected tissues.  

**Additional file 2:** **Supplementary Figure 1. A** PCR analysis of Jujube (diseased tissues),Paulownia arbuscular diseased tissues, and *N. indicum *tissues. well # 1:Ladder, 2: Jujube sample, 3: Paulownia arbuscular diseased sample, 4-7:diseased *N. indicum*, 8: healthy *N. indicum,* 9: Jujube sample, and10: Paulownia arbuscular diseased sample. **B** Nested PCR detection assay(M: Ladder, 1-4: diseased *N. indicum *tissues, 5: Paulownia arbusculardiseased tissues). **Supplementary Figure 2.**Summary of annotation; Number of *Nerium Indicum *L. genes annotated indifferent databases. **Supplementary Figure 3. **Scatter plots showing KEGG pathways in which thedifferentially expressed genes were enriched in NOHP vs NOWP and NOHS vs NOWS.NOHS, NOWS, NOHP, and NOWP represent non-infected stem, WBD infected stem,non-infected phloem, and WBD infected phloem, respectively. **Supplementary Figure 4.**Scatter plots showing KEGG pathways in which the specifically expressed geneswere enriched in A) NOHP, B) NOWP, C) NOHS, and D) NOWS. NOHS, NOWS, NOHP, andNOWP represent non-infected stem, WBD infected stem, non-infected phloem, andWBD infected phloem, respectively. **Supplementary figure 5. **OPLS-DA of the metabolites that weredifferentially accumulated between **A** NOHP vs NOWP and **B** NOHS vs NOWS. WhereNOWS, NOHS, NOWP, and NOHP represent infected stem tip, healthy stem tip,infected phloem, and healthy phloem of *N. indicum.*

## Data Availability

The raw transcriptome data have been submitted to NCBI SRA under the project number: PRJNA764871 (https://www.ncbi.nlm.nih.gov/bioproject/764871).

## Abbreviations

TIR1: Transport Inhibitor Response1
GH3: Glycoside hydrolase 3
CRE1: CYTOKININ RESPONSE 1
A-AAR1: Type-A Arabidopsis response regulator 1
AHP: Arabidopsis histidine phosphotransfer proteins
GID1: Gibberellin insensitive dwarf 1
CTR1: CONSTITUTIVE TRIPLE RESPONSE 1
EIN3: ETHYLENE INSENSITIVE 3
ERF1/2: Ethylene responsive factor 1/2
ETR: Ethylene receptor
SIMKK: Stress induced MAPK kinase kinase
EBF1/2: Ethylene binding factor 1/2
TCH4: Touch 4
CYCD3: Cyclin-D 3
BIN2: BR Insensitive 2
BZR1/2: BRASSINAZOLE-RESISTANT 1
JA: Jasmonic acid
COI1: CORONATINE INSENSITIVE 1
SA: Salicylic acid
SAR: System acquired resistance
PR-1: Pathogenesis related protein 1
ROS: Reactive oxygen species
TGA: TF TGA
Rboh: Respiratory burst oxidase
NOS: Nitric-oxide synthase
CNGCs: Cyclic nucleotide gated channel
FRK1: FLG22-induced receptor-like kinase 1
NHO1: Glycerol kinase
PTI5: Pto-interacting protein 1
MKK1/2: MAPK-kinase kinase 1/2
RAR1: Cysteine and histidine-rich domain-containing protein RAR1
EDS1: Enhanced disease susceptibility 1 protein
RPS2: Disease resistance protein RPS2
CYP2J: Cytochrome P450 family 2 subfamily J
CYP3A4: Cytochrome P450 family 3 subfamily A4
COR: Chloroplastic oxoene reductase
CCOAOMT1: Caffeoyl-CoA O-methyltransferase
4CL: 4-Coumarate-CoA ligase
Kat: Catalase-peroxidase
CCR: Cinnamoyl-CoA reductase
F5H: Ferulate-5-hydroxylase
PAL: Phenylalanine ammonia-lyase
CSE: Caffeoylshikimate esterase
CAD: Cinnamyl-alcohol dehydrogenase
SGtf: Scopoletin glucosyltransferase
RPN4: Regulatory Particle Non-ATPase 4
DEGs: Differentially expressed genes
DAMs: Differentially accumulated metabolites
NOHS: Stem tip from healthy N. indicum
NOWS: Stem tip from N. indicum with WBD symptoms
NOHP: Non-diseased N. indicum phloem
NOWP: Phloem from N. indicum with WBD symptoms
