# Supplementary material for: Combined transcriptome and metabolome analysis of Nerium indicum L. elaborates the key pathways that are activated in response to witches’ broom disease
Source: BMC Plant Biol. 2022 Jun 14;22:291. doi: 10.1186/s12870-022-03672-z (PMC9199210; doi:10.1186/s12870-022-03672-z)
Supplement: Supplementary file 2 — Additional file 2: Supplementary Figure 1. A PCR analysis of Jujube (diseased tissues),Paulownia arbuscular diseased tissues, and N. indicum tissues. well # 1:Ladder, 2: Jujube sample, 3: Paulownia arbuscular diseased sample, 4-7:diseased N. indicum, 8: healthy N. indicum, 9: Jujube sample, and10: Paulownia arbuscular diseased sample. B Nested PCR detection assay(M: Ladder, 1-4: diseased N. indicum tissues, 5: Paulownia arbusculardiseased tissues). Supplementary Figure 2.Summary of annotation; Number of Nerium Indicum L. genes annotated indifferent databases. Supplementary Figure 3. Scatter plots showing KEGG pathways in which thedifferentially expressed genes were enriched in NOHP vs NOWP and NOHS vs NOWS.NOHS, NOWS, NOHP, and NOWP represent non-infected stem, WBD infected stem,non-infected phloem, and WBD infected phloem, respectively. Supplementary Figure 4.Scatter plots showing KEGG pathways in which the specifically expressed geneswere enriched in A) NOHP, B) NOWP, C) NOHS, and D) NOWS. NOHS, NOWS, NOHP, andNOWP represent non-infected stem, WBD infected stem, non-infected phloem, andWBD infected phloem, respectively. Supplementary figure 5. OPLS-DA of the metabolites that weredifferentially accumulated between A NOHP vs NOWP and B NOHS vs NOWS. WhereNOWS, NOHS, NOWP, and NOHP represent infected stem tip, healthy stem tip,infected phloem, and healthy phloem of N. indicum. [file 12870_2022_3672_MOESM2_ESM.docx]

**Supplementary Figure 1.** A) PCR analysis of Jujube (diseased tissues), Paulownia arbuscular diseased tissues, and *N. indicum* tissues. well # 1: Ladder, 2: Jujube sample, 3: Paulownia arbuscular diseased sample, 4-7: diseased *N. indicum*, 8: healthy *N. indicum,* 9: Jujube sample, and 10: Paulownia arbuscular diseased sample. B) Nested PCR detection assay (M: Ladder, 1-4: diseased *N. indicum* tissues, 5: Paulownia arbuscular diseased tissues).

**Supplementary Figure 2.** Summary of annotation; Number of *Nerium Indicum* L. genes annotated in different databases.

**Supplementary Figure 3.** Scatter plots showing KEGG pathways in which the differentially expressed genes were enriched in NOHP vs NOWP and NOHS vs NOWS. NOHS, NOWS, NOHP, and NOWP represent non-infected stem, WBD infected stem, non-infected phloem, and WBD infected phloem, respectively.


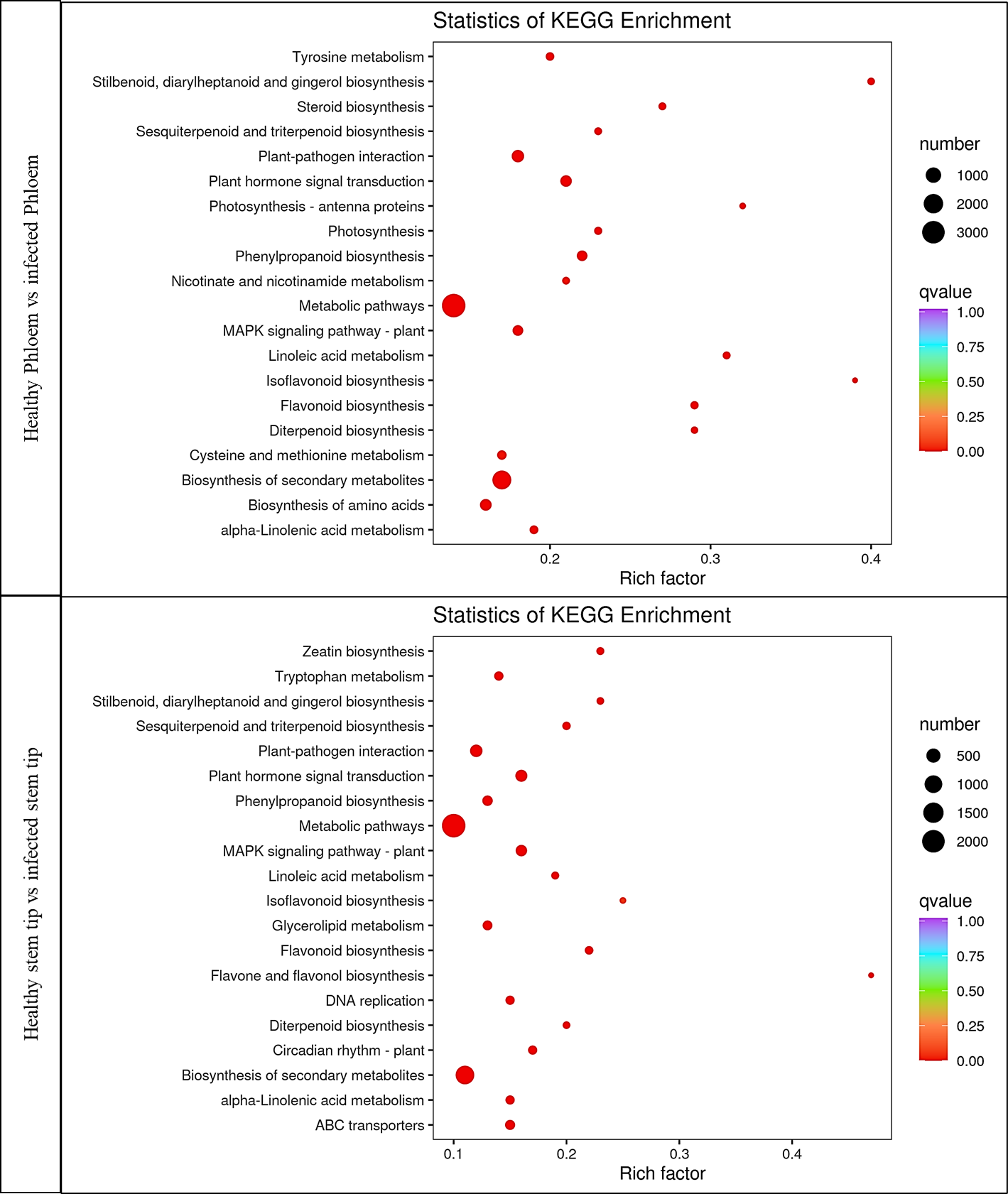


**Supplementary Figure 4.** Scatter plots showing KEGG pathways in which the specifically expressed genes were enriched in A) NOHP, B) NOWP, C) NOHS, and D) NOWS. NOHS, NOWS, NOHP, and NOWP represent non-infected stem, WBD infected stem, non-infected phloem, and WBD infected phloem, respectively.


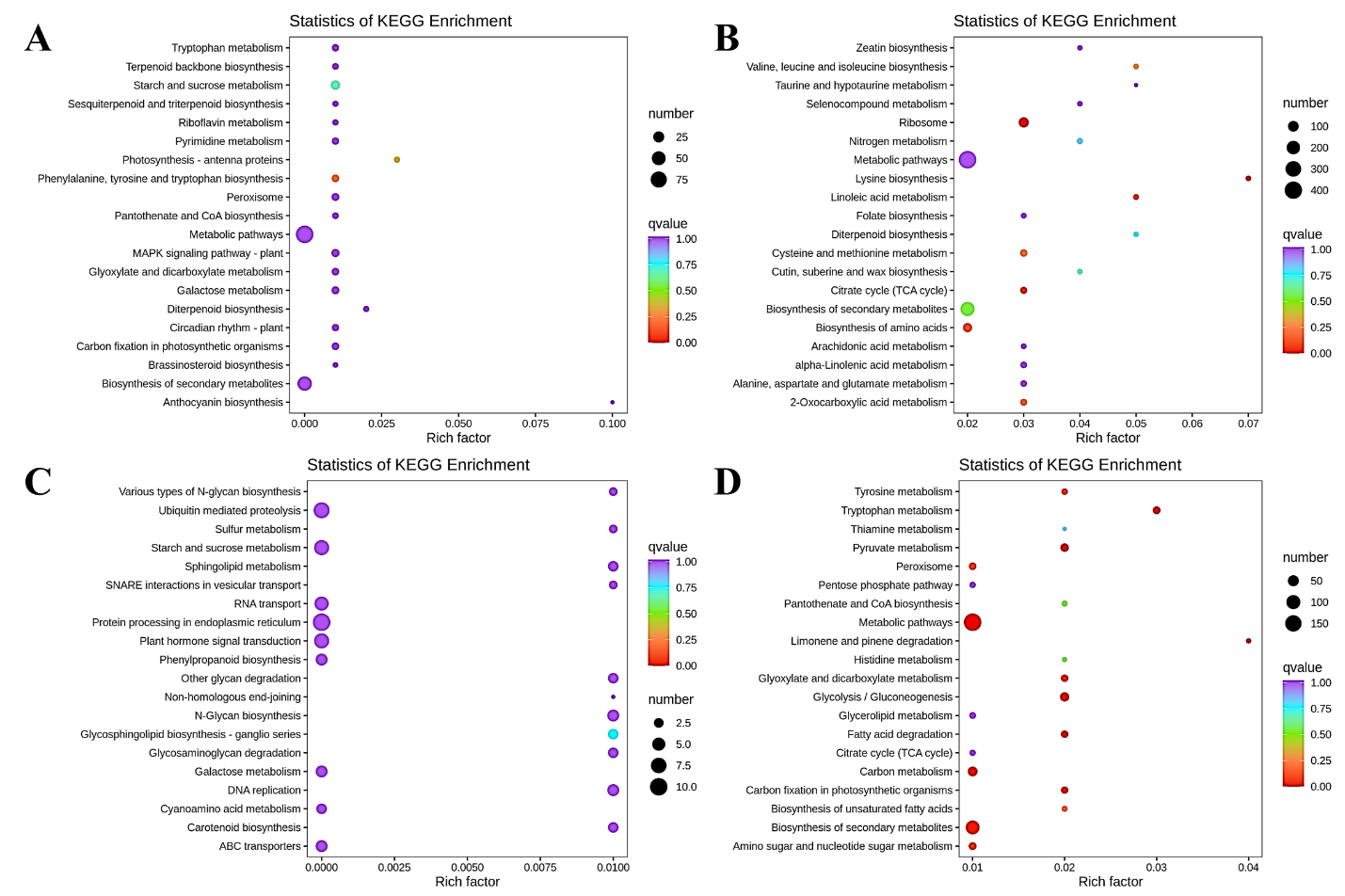


**Supplementary figure 5.** OPLS-DA of the metabolites that were differentially accumulated between A) NOHP vs NOWP and B) NOHS vs NOWS. Where NOWS, NOHS, NOWP, and NOHP represent infected stem tip, healthy stem tip, infected phloem, and healthy phloem of *N. indicum.*

**
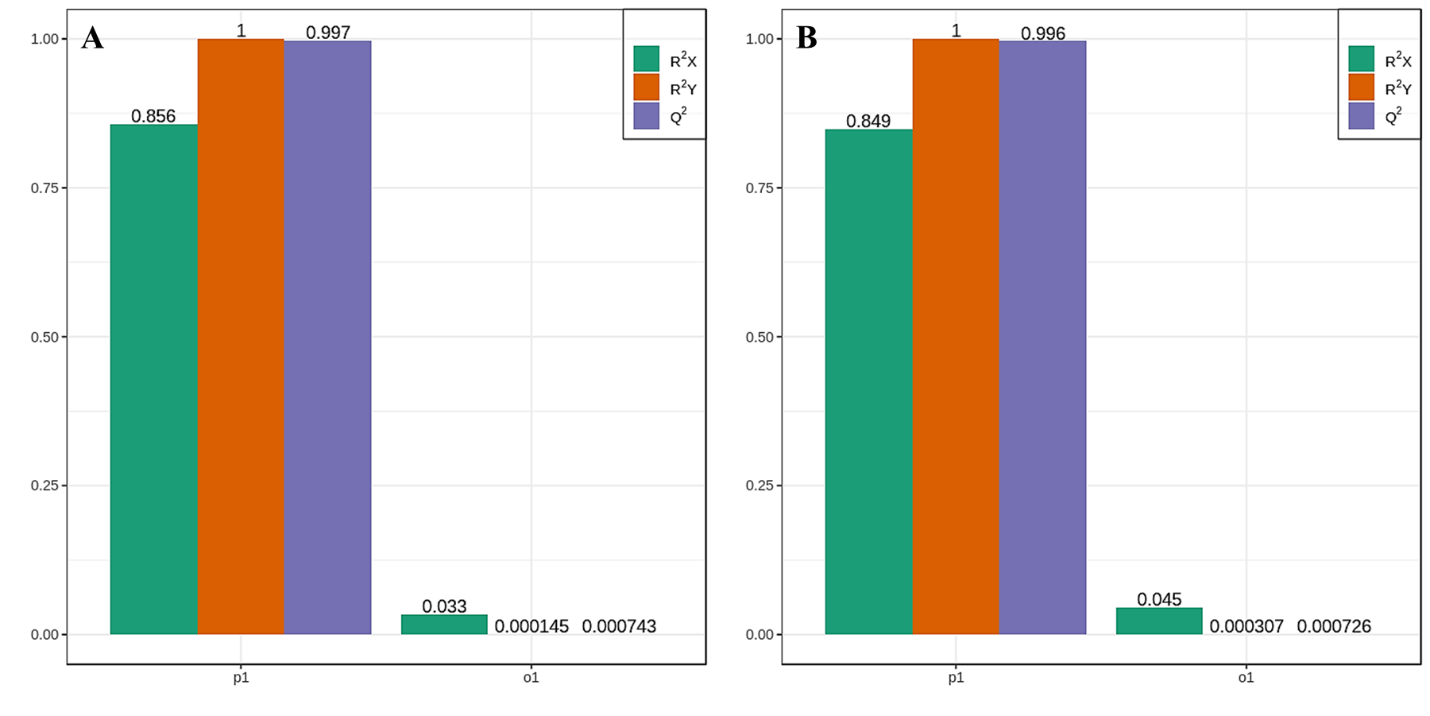
**
